# Bioassay-Guided Isolation and HPLC Quantification of Antiproliferative Metabolites from *Stahlianthus thorelii*

**DOI:** 10.3390/molecules25030551

**Published:** 2020-01-28

**Authors:** Nham-Linh Nguyen, Thanh-Hoa Vo, Yu-Chi Lin, Chia-Ching Liaw, Zhi-Hu Lin, Mei-Chuan Chen, Yao-Haur Kuo

**Affiliations:** 1 The Ph.D. Program in Clinical Drug Development of Herbal Medicine, College of Pharmacy, Taipei Medical University, Taipei 11031, Taiwan; nguyenlinhnham4201@gmail.com (N.-L.N.); hoavo0808@gmail.com (T.-H.V.); 2 Division of Chinese Materia Medica Development, National Research Institute of Chinese Medicine, Taipei 11221, Taiwan; m8952612@hotmail.com (Y.-C.L.); starccliaw@gmail.com (C.-C.L.); tiger77749@gmail.com (Z.-H.L.); 3 Graduate Institute of Natural Products, Kaohsiung Medical University, Kaohsiung 80708, Taiwan; 4 Department of Biochemical Science and Technology, National Chiayi University, Chiayi 60004, Taiwan; 5 Traditional Herbal Medicine Research Center of Taipei Medical University Hospital, Taipei 110, Taiwan; 6 Graduate Institute of Intergrated Medicine, College of Chinese Medicine, China Medical University, Taichung 40402, Taiwan

**Keywords:** *Stalianthus thorelii* Gagnep., Zingiberaceae, C-benzylated dihydrochalcone, cytotoxicity, antiproliferative activity

## Abstract

In folk medicine, *Stahlianthus thorelii* Gagnep. has been used to treat diseases related to inflammation, ulcers, and cancer. There are no reports concerning the chemical components and bioactivities of *S. thorelii*; thus, this study aims to explore the phytochemicals, quantify the main compounds, and test the anticancer activity of isolates from *S. thorelii.* Dried rhizomes were extracted with 95% ethanol and, then, partitioned, fractionated, and isolated. On the basis of the result of the antiproliferative activity of the fractions, seven isolates were yielded and were identified by spectroscopic analyses. The inhibition of cancer proliferation was determined by an MTT assay and the deployed IC_50_ to value their efficacy. Seven compounds containing one new C-benzylated dihydrochalcone derivative, thorechalcone A (**1**) and **2**–**7** were isolated from *S. thorelii.* In terms of the bioactivity, compounds **1** and **3** displayed promising antiproliferative activity (WiDr, A549, and HepG2), with IC_50_ values <40 µM. The HPLC-UV method of quantification of two major compounds (**3** and **4**) was also validated. This study presented the isolations of antiproliferative potentials of new chalcone and known flavonoid derivatives from S. *thorelii.* The validated simple, accurate, and rapid HPLC method could be deployed for the quality control of herbal drugs.

## 1. Introduction

Zingiberaceae is commonly known as a zinger family consisting of perennial herbs used in traditional medicines. This family is widely distributed in Asia, Africa, and America, comprising more than 1300 species and around 52 genera. Many species in this family have already been studied for their phytochemicals and bioactivities, which display significant anticancer, antimicrobial, anti-inflammation bioactivities [1]. The potential of anticancer activities of the species in the Zingiberaceae family have been were investigated through many studies [2,3,4,5,6,7,8,9]. Extracts of eight species of Zingiberaceae including *Amommum cardamomum, Curcuma longa, C. mangga, C. xanthorrhiza, Kaempferia pandurata, Zingiber officinale, Z. aromaticum,* and *Z. cassumunar* were found to inhibit the growth of MCF-7 and HT-29 cancer cell lines [2]. In 2015, an ethanolic extract of *C. longa* was reported to possess the antiproliferative and proapoptotic effects on murine melanoma B164A5 [3] and prostate cancer cell lines [4]. In addition, *Z. officinale* [5,6], *K. pandurata* [7], and *Alpinia galangal* [8] were mentioned to have the promising cytotoxicity against several cancer cell lines. Curcumin is a major ingredient extracted from Curcuma species, which had the antiproliferative activity and was proved to induce apoptosis of Rb cells via inhibition of JAK/STAT pathway [9].

*Stahlianthus thorelii* and *Stahlianthus invocratus* are members of the Zingiberaceae famil and both are known as “Khuong tam that” used in Vietnamese and Chinese folk medicine, respectively, to treat inflammation, pneumonia, diarrhea, and anticancer [10,11,12]. However, there are seldom reports concerning chemical components and bioactivities related to diseases for *Stahlianthus* species except for limited studies on *Stahlianthus invocratus* about its chemical components [10,13], and the bioactivities of its ethanol extract [14]. This study aimed to isolate bioactive compounds and elucidate their structures, based on antiproliferative-directed fractionations. Then, the main and active compounds were quantified by HPLC-UV. The method was validated for the quantification of two major active compounds. To the best of the authors’ knowledge, this is the first report about *Stahlianthus thorelii* concerning both phytochemistry and anticancer potential.

## 2. Results and Discussion

### 2.1. Bioactivity-Guided Isolation of Chemical Constituents 

A comparison with the other three solvents extracted layers (SEA, SBU, and SW) showed that the EtOAc layer (SEA) had the most potent antiproliferative activities. Therefore, SEA was selected to further make fractionations and subfractions. SF3, SF7, and SF9 showed the inhibition of human colon adenocarcinoma (WiDr) proliferation with IC_50_ = 43.42, 25.49, and 20.04 µg/mL, respectively (Table 1). Furthermore, SF7 and SF9 also displayed antiproliferative activities in cell lines A549, MCF-7, and HepG2. SF3 was fractionated to simple subfractions; and SF 3.5 and SF 3.6 had the same antiproliferative activity in the WiDr cell lines. These two subfractions also displayed the antiproliferation of A549, MCF-7, and HepG2, despite SF3 having a weak effect on those cell lines.

**Figure 1:**
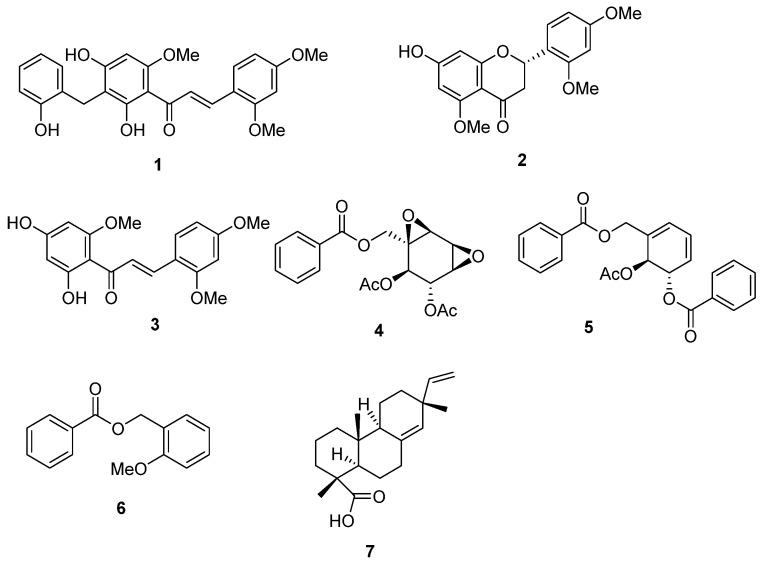
Structures of isolates from *Stahlianthus thorelii.*

In this way, the bioactivity-guided fractionation of *S. thorelii* extracts led to isolating one new compound, thorechalcone A (**1**), together with six known compounds, **2**–**7** (Figure 1).

### 2.2. Structural Determination

The structures of the isolates were determined by the analysis of their spectral data (^1^H-NMR, ^13^C-NMR, DEPT, HSQC, HMBC, ^1^H-^1^H COSY, ultraviolet, infrared, and mass spectrometry). 

**Figure 2:**
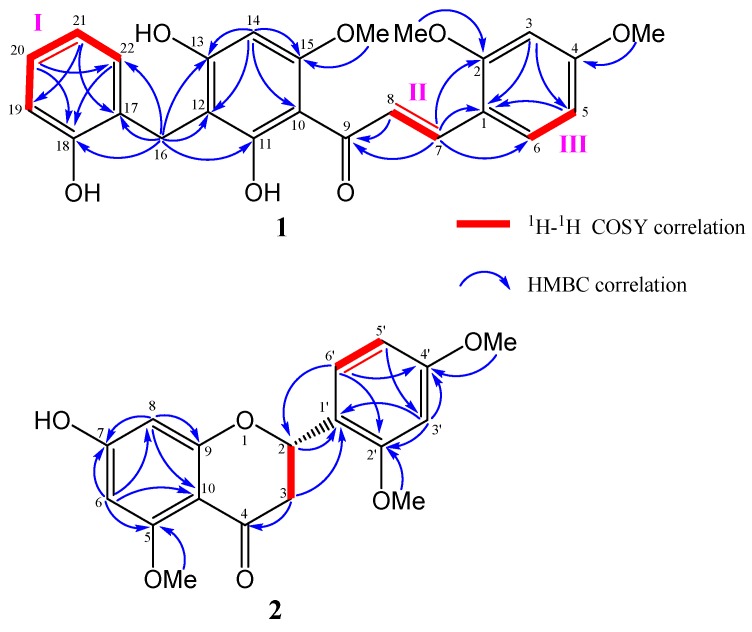
Key HMBC and ^1^H-^1^H COSY correlations of compounds **1** and **2**.

**Figure 3:**
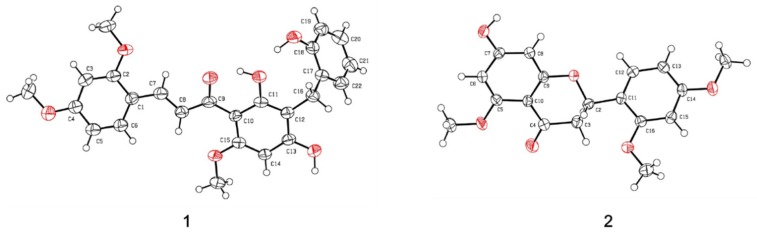
ORTEP diagram showing the crystallographic atom-numbering scheme and solid-state conformation of compounds **1** and **2**.

The HR-ESI-MS of compound **1** showed a pseudo-molecular ion peak at *m/z* 435.1444 [M − H]^−^, consistent with the molecular formula of C_25_H_24_O_7_ (Calcd. for C_18_H_23_O_7_, 435.1438), containing 14 degrees of unsaturation. The IR spectrum revealed the absorptions of hydroxyl (3331 cm^−1^), and aromatic (1603, 1455 cm^−1^) functions. The UV absorption bands at 285 nm suggested that **1** possessed a benzaldehyde or acetophenone functional group. The ^13^C-NMR and DEPT spectra revealed that compound **1** was categorized into three methoxyl carbons at δ_C_ 56.1, 56.2, and 56.9; one methylene carbon at δ_C_ 23.2; 10 methine carbons at δ_C_ 92.5, 99.1, 107.0, 116.2, 120.3, 126.0, 127.7, 131.2, 131.5, and 138.5; as well as 11 quaternary carbons at δ_C_ 105.9, 108.2, 118.1, 128.2, 155.6, 161.2, 162.6, 164.1, 164.4, 166.4 and 193.5. Three sections (I–III, Figure 2) were established from key cross-peaks in the ^1^H-^1^H COSY spectrum (H-5/H-6, H-7/H-8, and H-19/H-20/H-21/H-22). In the HMBC spectrum, on the one hand, the cross-peaks of H-16 (δ_H_ 3.89) with C-11 (δ_C_ 166.4), C-12 (δ_C_ 108.2), C-13 (δ_C_ 164.4), C-17 (δ_C_ 128.2), C-18 (δ_C_ 155.6), and C-22 (δ_C_ 131.5) and that of H-14 (δ_H_ 6.20) with C-10 (δ_C_ 147.7), C-12, C-13, and C-15 (δ_C_ 145.9) revealed that compound **1** possessed a diphenylmethane structure (Figure 2). On the other hand, the correlations between H-7 (δ_H_ 8.02) and C-1 (δ_C_ 118.1), C-2 (δ_C_ 161.2), C-6 (δ_C_ 131.2, and C-9 (δ_C_ 193.5), as well as between H-3 (δ_H_ 6.64) with C-1 and C-5 (δ_C_ 107.0) allowed a cinnamaldehyde unit in **1**. Additionally, three methoxy groups were substituted at the C-2, C-4, and C-15 positions due to their respective HMBC correlations. Moreover, the long-range correlations between H-8 and C-9 and C-10 decided the linkage of diphenylmethane and cinnamaldehyde units. The established structure of **1** as above was also consistent with a single-crystal X-ray diffraction analysis showing a perspective drawing, as shown in Figure 3. Accordingly, the structure of *C*-benzylated dihydrochalcone derivative (**1**) was determined as (*E*)-1-(3,5-dihydroxy-4-(2-hydroxybenzyl)-2-methoxyphenyl)-3-(2,4-dimethoxyphenyl) prop-2-en-1-one and was named thorechalcone A. 

Compound **2** (7-hydroxy-5,2′,4′-trimethoxyflavanone) was obtained as an amorphous solid with the molecular formula C_18_H_20_O_7_ as analyzed by HRESIMS and had nine degrees of unsaturation. The IR spectrum exhibited absorption bands at 1584 and 1464 cm^−1^ for benzyl and strong absorption bands at 3417 cm^−1^ for hydroxy together with the UV bands at 230, 250, and 284 nm, which indicated the flavanone skeleton of **2**. The ^13^C-NMR spectrum of **2** showed 18 carbon signals including eight quaternaries (including one conjugated carbonyl carbon at δ_C_ 187.9), six methines, one methylene, and three methoxyl (δ_C_ 55.3, 55.6, and 55.7) carbons via the ^1^H and ^13^C-NMR and HSQC correlation analyses. The proton signals at δ_H_ 5.55 (1H, dd, *J* = 12.5, 2.5 Hz), 2.95 (1H, dd, *J* = 16.5, 12.5 Hz), and 2.67 (1H, dd, *J* = 16.5, 2.5 Hz), and the corresponding carbon signals at δ_C_ 73.0 and 44.0, respectively, were characteristics of the oxygenated methine and methylene groups of a flavanone unit. Moreover, five aromatic protons were evident in the ^1^H-NMR spectrum, with splitting patterns characteristic of a 1,2,4-trisubstituted aromatic ring (δ_H_ 6.57, 1H, dd, *J* = 8.0, 2.0 Hz; 6.61, 1H, d, *J* = 2.0 Hz; and 7.38, 1H, d, *J* = 8.0 Hz), and two protons were assigned to H-6 (δ_H_ 5.95) and H-8 (δ_H_ 6.06) with w-coupling (*J* = 2.5 Hz). The A and B rings of the flavanone framework were established by the HMBC correlation of H-3 with C-2, C-4, C-1′; H-6′ with C-2, C-4′, C-2′; H-6 with C-5, C-7, C-10; and H-8 with C-7, C-9, C-10 (Figure 2). The chemical shift of C-4 at δ_C_ 187.9, together with the long-range heteronuclear correlations, indicated the existence of conjugated carbon at C-5 with a methoxyl group, and the other methoxyl groups were substituted at the C-2′ and C-4′ positions of the B ring. The planar structure of **2** was also confirmed by single-crystal X-ray diffraction analysis (Figure 3). Moreover, the *S* configuration of C-2 was deduced based on the CD spectrum of **2** exhibiting negative Cotton effects at 285 nm. Thus, the structure of **2** was elucidated as *S*-2-(2,4-dimethoxyphenyl)-7-hydroxy-5-methoxychroman-4-one, possessing the same structure as the reported cerasinone [15] as assigned only by the ^1^H-NMR data. However, additional spectroscopic evidence, including the ^13^C-NMR (Table 2) and X-ray diffraction data mentioned above were also provided here.

Compound **3** [(2*E*)-1-(2,4-Dihydroxy-6-methoxyphenyl)-3-(2,4-dimethoxyphenyl)-2-propen-1-one] was obtained as an amorphous yellow solid C_18_H_18_O_6_; ESIMS *m/z* 330 [M − H]^−^. ^1^H-NMR (500 MHz, DMSO) δ_H_: 7.89 (d, *J* = 16 Hz, 1H); 7.83 (d, *J* = 16 Hz, 1H); 7.63 (d, *J* = 9 Hz, 1H); 6.65 (d, *J* = 2.5 Hz, 1H); 6.62 (dd, *J* = 2.5, 8 Hz, 1H); 6.00 (d, *J* = 2 Hz, 1H); 5.90 (d, *J* = 2.5 Hz, 1H); 3.90 (s, 3H); 3.87 (s, 3H); 3.84 (s, 3H); 3.08 (s, 2H). ^13^C-NMR (125 MHz, DMSO) δ_C_: 191.8, 166.4, 164.6, 162.8, 162.6, 159.9, 137.6, 130.6, 124.8, 116.3, 106.4, 105.1, 98.4, 95.9, 91.6, 55.9, 55.8, 55.5, The data were confirmed by the further comparison of the NMR data with those in the literature [16].

Compound **4** [(+)-Crotepoxide] was obtained as an amorphous white solid C_18_H_18_O_8;_ ESIMS *m/z* 362 [M + Na]^+^. ^1^H-NMR (500 MHz, DMSO) δ_H_: 7.98 (d, *J* = 10.5 Hz, 2H); 7.69 (t, *J* = 9.5 Hz, 1H); 7.55 (t, *J* = 10 Hz, 1H); 5.74 (d, *J* = 2.5 Hz, 1H); 6.62 (dd, *J* = 2.5, 8 Hz, 1H); 6.00 (d, *J* = 2 Hz, 1H); 5.90 (d, *J* = 2.5 Hz, 1H); 3.90 (s, 3H); 3.87 (s, 3H); 3.84 (s, 3H); 3.08 (s, 2H). ^13^C-NMR (125 MHz, DMSO) δ_C_: 191.8, 166.4, 164.6, 162.8, 162.6, 159.9, 137.6, 130.6, 124.8, 116.3, 106.4, 105.1, 98.4, 95.9, 91.6, 55.9, 55.8, and 55.5. The data were confirmed by the further comparison of the NMR data with those in the literature [17].

Compound **5** [(−)-1,6-Desoxytingtanoxide] was obtained as an amorphous yellow oil C_23_H_20_O_6;_ ESIMS *m/z* 392 [M + Na]^+^. ^1^H-NMR (500 MHz, CDCl_3_) δ_H_: 8.05 (dd, *J* = 1, 8.5 Hz, 2H); 7.98 (dd, *J* = 1.5, 8 Hz, 2H); 7.58 to 7.53 (m, 2H); 7.42 (t, *J* = 10 Hz, 2H); 7.38 (t, *J* = 8 Hz, 2H); 6.32 (d, *J* = 5.5 Hz, 1H); 6.20 (dd, *J* = 5, 10 Hz, 1H); 6.06 (dd, *J*= 4.5, 9.5 Hz, 1H); 6.02 (d, *J*= 6.5 Hz, 1H); 5.74 (ddd, *J*= 6, 4, 1.5 Hz, 1H); 4.94 (d, *J*= 3 Hz, 2H), 2.03 (s, 3H). ^13^C-NMR (125 MHz, CDCl_3_) δ_C_: 170.5, 166.5, 166.0, 133.6, 133.6, 131.6, 130.3, 130.2, 130.1, 128.8, 128.8, 126.4, 126.0, 125.5, 71.7, 70.1, 65.1, 21.3. The data were confirmed by the further comparison of the NMR data with those in the literature [18].

Compound **6** (*O*-Methoxybenzoyl benzoate) was obtained as an amorphous yellow solid C_15_H_14_O_3;_ ESIMS *m/z* 242 [M + Na]^+^. ^1^H-NMR (500 MHz, CDCl_3_) δ_H_: 8.11 (dd, *J* = 8.5, 1 Hz, 2H); 7.56 (t, *J* = 7.5 Hz, 1H); 7.44 to 7.48 (m, 3H); 7.35 (td, *J* = 7.5, 1.5 Hz, 1H); 7.0 (td, *J* = 1, 7.5 Hz, 1H); 6.94 (d, *J* = 8 Hz, 1H); 5.45 (s, 2H); 3.89 (s, 3H); ^13^C-NMR (125 MHz, CDCl_3_) δ_C_: 166.6, 157.5, 132.9, 130.5, 129.7, 129.4, 128.3, 124.5, 120.5, 110.5, 62.2, 55.5. The data were confirmed by the further comparison of the NMR data with those in the literature [19].

Compound **7** (Sandaracopimaric acid) was obtained as an amorphous yellow solid C_20_H_30_O_2;_ ESIMS *m/z* 302 [M − H]^−^. ^1^H-NMR (500 MHz, acetone-*d*_6_) δ_H_: 5.77 (dd, *J* = 14, 9 Hz, 1H); 5.23 (s, 1H); 4.9 (dd, *J* = 14.5, 1.5 Hz, 1H); 4.86 (dd, *J* = 9, 1Hz, 1H); 2.24 (ddd, *J* = 2, 4, 11.5 Hz, 1H); 2.09 (m, 1H); 1.97 (d, *J* = 10.5, 2 Hz, 1H); 1.82 (s, 3H); 1.79 (s, 3H); 1.78 (s, 3H); 1.63 (overlap)1.56 (overlap), 1.47 (overlap), 1.44 (dd, *J*= 10.5, 3.5 Hz, 1H), 1.36 (ddd, *J* = 3, 9, 11 Hz, 1H), 1.28 (m, 1H), 1.19 (s, 3H), 1.16 (td, *J* = 3, 11 Hz, 1H), 1.04 (s, 3H), 0.87 (s, 3H). ^13^C-NMR (125 MHz, acetone-*d*_6_) δ_C_: 179.1, 148.7, 136.7, 128.8, 109.7, 50.7, 48.9, 46.7, 38.3, 37.6, 37.2, 36.9, 35.4, 34.4, 25.5, 24.7, 18.4, 18.0, 16.6, and 14.7. The data were confirmed by the further comparison of the NMR data with those in the literature [20].

The ^13^C-NMR, ^1^H-NMR and ESIMS data of **1**–**7** are shown in the supplementary section as Figures S1–S22.

### 2.3. Evaluation of the Anticancer Potential of Isolated Compounds

In the present study, an MTT cell proliferation assay was deployed for assessing the cytotoxicity of the fractions and isolated compounds. The high level of MTT reduction correlated to the high proliferation of cancer cells. The number of viable cells led to a large amount of formazan, which was created in a culture. Mitomycin *c*, which is a positive control, was used as a reference compound for all the cytotoxic assays in this study. 

As shown in Table 3, the cytotoxicity of compounds **1**–**6** was tested on four cancer cell lines (A549, WiDr, HepG2, and MCF-7) by the MTT assay. The compounds **1** and **3** exhibited stronger cytotoxic activities on cell lines A549, WiDr, and HepG2, due to their possessing a chalcone structure. Interestingly, compound **1** had more potent cytotoxicity against the three cell lines than that of **3**, based on the additional *o*-hydroxybenzyl moiety in structure **1**. Although the remaining compounds **4**–**6** displayed moderate activities on different cell lines, this study was the first to report antiproliferation on four tumor cell lines for **4** and **5**. In previous reports, **4** ((+)-crotepoxide) showed antimutagenicity in a Vitotox assay [17] and 1,6-deoxytingtanoxide (**5**) displayed mild cytotoxicity against human pancreatic cancer cell lines [21]. The bioassay data showed that compound **6** (*O*-methoxybenzoyl benzoate) did not have significant cytotoxicity on MCF-7; this result correlated with a previous study [22].

### 2.4. Quantitative Analysis of Compounds ***3*** and ***4***

The validated method was applied to the quantitative analysis of bioactive compound **3** and major yielded compound **4** from the *S. thorelii* rhizome. It was considered that 210 nm could be best used to analyze the profile of the compounds after comparing the record of the chromatograms of the extract solution running at wavelengths within 200 to 550 nm. Good linearity was obtained for each of constituents (R^2^ > 0.995). The titled plant sample, *Stahlianthus thorelii*, was collected from three different areas in December 2018 (shown in Table 4). The quantitation was determined with three preparative samples and was analyzed three times. The concentrations were detected based on a linear regression and the average contents were obtained (*p* < 0.01). The contents of compounds **3** and **4** in the *S. thorelii* rhizome were 0.106% and 0.013%, respectively.

### 2.5. Validation of the HPLC-UV Analysis Method

To evaluate the quality of *S. thorelii*, two major compounds, **4** and anticancer **3,** were retained for their quantification in the plant material. The HPLC-UV method was validated in terms of the analysis of the system suitability, specificity, precision, accuracy, linearity, and limits of detection and quantification. The validation summary is found in Table 5.

According to ICH guidelines, system suitability testing is based on the concept that the equipment, electronics, analytical operations, and samples to be analyzed constitute an integral system that can be evaluated as such. Testing is checked by calculating the retention time (t_R_), peak area (A), theory plate (N), and resolution (Rs) factors. A residual sum of the squares of all the calculated parameters that is less or more than 2%, is within the acceptable limits, indicates good selectivity of the method, and ensures system performance. The linearity of this method was confirmed by a linear regression function. The calculations were based on seven different concentrations (*n* = 7). The regression equations were calculated using y = 32638x (R^2^ = 0.9956) and y = 57440x (R^2^ = 0.9969) for compounds **4** and **3**, respectively, as illustrated in Table 5.

The limit of detection (LOD) and limit of quantification (LOQ) were determined by calculating them as three and 10 times the intensity of the baseline noise, respectively. The LOD and LOQ of compound **4** were 0.05 and 0.17 µg/mL, respectively, and the LOD and LOQ of compound **3** were 0.025 and 0.08 µg/mL, respectively. The specificity of the method was investigated. Specificity represents the ability to assess an analyte unequivocally in the presence of components which may be expected to be present by comparing the retention time and the UV spectra with the standard. As can be seen from Figure 2, the retention times of the compound **4** peak (24.8 min) and the compound **3** peak (30.0 min) in the sample were the same as the peaks in the standard. In addition, the peaks in the sample separated absolutely, and the peak area went up when adding the standards into the sample. Whereas, in the blank sample, there was no peak in the retention time of compound **4** and compound **3**. This result indicated that the method could be used to analyze compound **4** and compound **3**. Furthermore, the solvent did not influence the major compound peaks, and PAD purity studies confirmed the purity of the investigated peaks.

The repeatability was determined using six samples (*n* = 6) by calculating the retention time and the peak area. The RSDs of all the calculated parameters were less than or equal to 2% (retention time) and 5.3% (peak area). The accuracy was calculated based on the recovery concentration in three levels. The amount of the standard was put into the powdered rhizome at a ratio of 80%, 100%, and 120% of the content of compounds **4** and **3** in the sample. According to the calculated concentrations of compounds **4** and **3**, the percentage of recovery of these compounds could be determined and the recovery parameters were less than or equal to 5.3%.

## 3. Materials and Methods

### 3.1. General Experimental Procedures

The ESI-MS data were obtained on a VQ Quattro 5022 mass spectrometer (VG-Biotech PVT). The HR-ESI-MS data were measured on a Finnigan MAT-95XL mass spectrometer. The NMR spectra were recorded on Bruker AVANCE400 (Burker Co., Rheinstetten, Germany) (400 MHz), Varian Unity Inova 500 MHz FT-NMR, and Varian VNMRS 600 spectrometers. (Palo Alto, CA, USA). A Sephadex LH-20 (GH Healthcare) (Chicago, IL, USA) Strata C18-E (55 μM, 70 Å) 10 g/60 mL, and Giga Tubes (Phenomenex) (Torrance, CA, USA) were used for column chromatography. HPLC was conducted on a Shimadzu LC-6AD series apparatus with an SPD-6AV UV-VIS detector that was equipped with a preparative Cosmosil 5C_18_ AR-II column (2.0 mm I.D. × 250 mm, 5 μM) (Nacalai Tesque, Inc., Kyoto, Japan). The single-crystal X-ray diffraction measurements were carried out on a Brucker D8 Venture Dual X-ray Single Crystal Diffractometer.

### 3.2. Plant Material

The plant material of *S. thorelii* was collected in An Giang Province, in the south of Vietnam, in 2017. The material was identified by the Faculty of Pharmacy, University of Medicine and Pharmacy at Ho Chi Minh City, where the voucher specimen (STV-20170224) was deposited. The rhizomes were dried and ground.

### 3.3. Extraction and Isolation

Air-dried rhizoma of *S. thorelii* (15 kg) were extracted three times using 95% ethanol (40 L) at 40 °C for 24 h each time. The extract was evaporated under reduced pressure to get the crude extract (500 g). Next, the extracted ethanol of *S. thorelii* was suspended and dissolved in 500 mL of H_2_O and partitioned continually by ethyl acetate and *n*-butanol (3 × 800 mL). After evaporation in a vacuum, fractions were prepared sequentially by ethyl acetate fraction (SEA) (120 g, 24%), *n*-butanol fraction (SBU) (40.5 g, 8.1%), and aqueous fraction (SW) (320 g, 64%) from the ethanol extracts. 

On the basis of the results of the cytotoxic assay, SEA (20 g) was selected to further isolate the active compounds. Furthermore, SEA was subjected to Sephadex (500 g) and eluted with methanol with a flow rate of 4.0 mL/min. All nine subfractions were collected by testing in TLC, and three compounds, **4** (74.0 mg), **1** (22.0 mg), and **3** (42.0 mg), were isolated in the precipitate form from subfraction SF3, and two potent cytotoxic subfractions, SF9 and SF7, respectively. SF7 (112 mg) was subjected to the reversed-phase preparative HPLC (Cosmosil 5C_18_ AR-II column; 2.0 mm I.D. × 250 mm, 5 μM, (Nacalai Tesque, Inc., Kyoto, Japan). with a flow rate of 10 mL/min, and 55% acetonitrile was used as a solvent system. Subfractions were yielded at 210 nm, and compound **2** (t_R_ = 8.36 min, 2.0 mg) was collected along with compound **3** (t_R_ = 20.13 min, 36.0 mg). SF3 (3.2 g) was separated by a C_18_ gel flask column eluted with acetonitrile (ACN)/H_2_O (1:9 to 7:3) and then a reversed-phase semi-preparative HPLC (Cosmosil 5C_18_ AR-II column; 2.0 mm I.D. × 250 mm, 5 μm, (Nacalai Tesque, Inc., Kyoto, Japan). was used to yield compound **7** (t_R_ = 82.52 min, 1.2 mg) from SF3.6 with 65% ACN under UV 210 nm. Using the same condition of semi-preparative HPLC as SF3.6, compounds **6** (t_R_ = 52.14 min, 2.0 mg) and **5** (t_R_ = 74.52 min, 17.0 mg) were yielded from SF 3.5. 

Thorechalcone A (**1**) A white amorphous powder; $[\alpha {]}_{D}^{25}$−1.4 (*c* 0.1, MeOH); UV (MeOH) λ_max_ 193, 285, 383 nm. IR (KBr) *ν*_max_ 3331, 1602, 1536, 1454, 1223, 1108, and 1026 cm^−1^; ^1^H and ^13^C-NMR spectroscopic data (Acetone-*d*_6_) are shown in Table 1; HR-ESI-MS *m/z* 435.1444 [M − H]^−^ (calcd. for C_25_H_23_O_7_, 435.1438).

### 3.4. X-ray Crystallographic Analysis

*Thorechalcone A* (**1**) A colorless crystal (0.64 × 0.09 × 0.01 mm^3^) was obtained by simple evaporation from an ACN solution. The crystal data were as follows: C_25_H_24_O_7_, orthorhombic, *a* = 16.253(3) Å, *b* = 8.3312(12) Å, *c* = 15.726(3) Å, *V* = 2094.7(6) Å^3^, space group P21/c, *Z* = 4, D_calcd_ 1.384 Mg/m^3^, λ = 0.71073 Å, absorption coefficient 0.101 mm^−1^, F(000) = 920, and T = 200(2) K. A total of 34,249 reflections were collected, of which 3697 independent reflections [R_int_ = 0.1865] with *I* > 2*σ (I)* were used for the analysis. The data were solved using the direct method, and the structure was refined by full-matrix least-squares on F^2^ values. All non-hydrogen atoms were refined with anisotropic thermal parameters. The hydrogen atom positions were idealized geometrically and allowed to ride on their parent atoms. The final indices were *R1* = 0.0593, *wR2* = 0.1337 with goodness-of-fit = 1.056. The final X-ray model is shown in Figure 3. The crystallographic data of compound **1** were deposited in the Cambridge Crystallographic Data Centre (CCDC), and the CCDC deposition number is CCDC 1948088.

*7-Hydroxy-5,2′,4′-trimethoxyflavanone* (**2**) A colorless crystal (0.79 × 0.05 × 0.02 mm^3^) was obtained by simple evaporation from an MeOH solution. The crystal data were as follows: C_18_H_18_O_6_, orthorhombic, *a* = 4.9370(2) Å, *b* = 14.8773(7) Å, *c* = 21.5417(11) Å, *V* = 1582.22(13) Å^3^, space group P212121, *Z* = 4, D_calcd_ 1.387 Mg/m^3^, λ = 0.71073 Å, absorption coefficient 0.104 mm^−1^, F(000) = 696, and T = 200(2) K. A total of 8424 reflections were collected, of which 2756 independent reflections [R_int_ = 0.0625] with *I* > 2*σ (I)* were used for the analysis. The data were solved using the direct method, and the structure was refined by full-matrix least-squares on F^2^ values. All non-hydrogen atoms were refined with anisotropic thermal parameters. The hydrogen atom positions were idealized geometrically and allowed to ride on their parent atoms. The final indices were *R1* = 0.0405, *wR2* = 0.0853 with goodness-of-fit = 1.062. The final X-ray model is shown in Figure 3. The crystallographic data of compound **2** were deposited in the Cambridge Crystallographic Data Centre (CCDC), and the CCDC deposition number is CCDC 1949802.

### 3.5. Colorimetric MTT In Vitro Assay

An MTT assay was employed to evaluate the proliferation of a number of cancer cell lines. Human lung carcinoma (A549), human breast adenocarcinoma (MCF-7), human colon adenocarcinoma (WiDr), and human hepatocellular carcinoma (Hep G2) were cultured in 75-T flasks with MEM supplemented with 5% FBS and a 1% penicillin-streptomycin antibiotics suspension. The cell suspensions after trypsinizing were seeded in 96-well plates at densities of 3000 cells per well. The 96-well plates were then incubated at 37 °C/5% CO_2_ to adhere the cell to the surface of the plate. After reaching the confluence (80%), the cells were treated with 200 µL medium containing 20 to 80 µg/mL of the extract(s) and the fraction(s), and 1 to 40 µg/mL of the pure compound(s), and incubated for 72 h before reading the results. Later, the medium was removed by vacuum suctioning and 200 µL of 0.2 mg/mL MTT was added for 4 h at 37 °C. At the end of the incubation, the MTT was discharged, and 200 µL of dimethyl sulfoxide (DMSO) was added into 96-well plates. The absorbance of the solution was measured at 570 nm by a microplate reader (Dynatech, MR 7000) (Dynatech Labs, Chantilly, VA, USA). The same procedure was conducted with mitomycin c (positive control) at a concentration of 0.4, 0.2, and 0.1 µg/mL. The mean IC_50_ was the inhibitor concentration, which reduced the proliferation of cells by 50%; this value was determined under the experimental conditions and calculated by the average of at least three independent tested results. 

The cell viability was measured using this equation:

(1) $$\%\;\mathrm{Inhibition}=\frac{\mathrm{Average}\;\mathrm{OD}\;\left(\mathrm{control}\right)-\mathrm{Average}\;\mathrm{OD}\;\left(\mathrm{treated}\right)}{\mathrm{Average}\;\mathrm{OD}\;\left(\mathrm{control}\right)}\;\times \;100$$

### 3.6. HPLC-SPD Separation Profile for Analysis of Seven Isolates from S. thorelii

**Figure 4:**
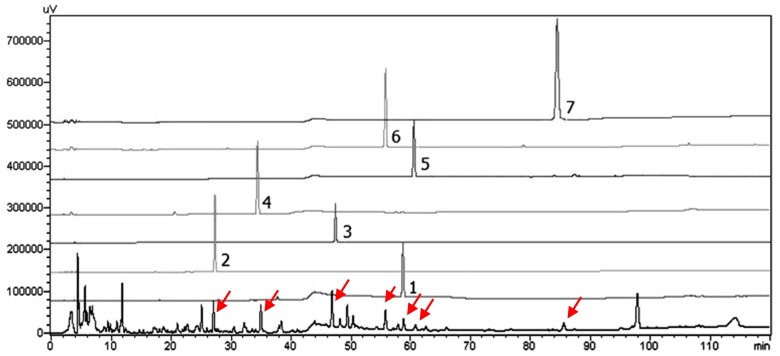
HPLC diagram of isolates from *S. thorelii* extract. (1) Thorechalcone A-t_R_ = 59 min, (2) cerasinone-t_R_ = 27.2 min, (3) [(2*E*)-1-(2,4-dihydroxy-6-methoxyphenyl)-3-(2,4-dimethoxyphenyl)-2-propen-1-one]-t_R_ = 46.5 min, (4) crotepoxide-t_R_ = 35 min, (5) (−)-1,6-desoxytingtanoxide-t_R_ = 61 min, (6) (*O*-methoxybenzoyl benzoate)-t_R_ = 55 min, and (7) sandaracopimaric acid t_R_ = 85min.

The HPLC-SPD analysis was performed using a 250 × 4.6 nm i.d., 5 µM, Cosmosil C_18_ column with an LC-20AT pump and an SPD-10A diode array detector (Shimazu). (Shimadzu, Kyoto, Japan). The mobile phase flow rate and the injection volume were 0.8 mL/min and10 µL, respectively. The wavelengths were set at 203 and 254 nm. The mobile phase was comprised of H_2_O (A) and ACN (B) and used a gradient program of separation condition as: 255 to 35% B from 0 to 15 min, 35% to 40% B from 15 to 20 min, 40% to 45% B from 20 to 30 min, 45% to 50% B from 30 to 35 min, 50% to 60% B from 35 to 45 min, 60% to 65% B from 45 to 55 min, 65% to 75% B from 55 to 85 min, and 75% to 100% B from 85 to 100 min (Figure 4).

### 3.7. Quantitation of Bioactive Compound ***3*** and Major Yielded Compound ***4***

Concentrated solutions of two standards (**3** and **4**) were prepared in the ACN, each at 1000 mg/L. After diluting the stock solutions with ACN to obtain the developing solutions, seven concentration levels were found in the range of 2.5–500 mg/L. The worked solutions were filtered by a PVDF filter (0.45 µM, Millipore) before HPLC injection. Linear regression analysis was applied to achieve linearity by the integrated peak areas (Y) vs. the concentration of each standard (X, mg/L) at five different concentrations.

Analysis conditions: 35% to 40% B from 0 to 15 min, 40% to 60% B from 15 to 20 min, 60% B from 20 to 25 min, 60% to 70% B from 25 to 35 min, and 70% to 80% B from 35 to 60 min.

#### 3.7.1. Validation of the HPLC-UV Method 

According to the ICH harmonized tripartite guidelines, validation of the analytical procedure included system suitability, accuracy, repeatability, specificity, detection limit, quantitation limit, and linearity. Samples, calibration, and validation standards were prepared separately.

#### 3.7.2. Preparation of Calibration Standards

This study measured 10.0 mg of compounds **3** and **4,** respectively, into a volumetric flask and diluted them to 10.0 mL with MeOH. Next, 250 µL of each solution were placed into a volumetric flask and diluted to 10.0 mL with MeOH. The final concentrations for compound **4** and compound **3** were 250 µg/mL and 55 µg/mL, respectively. Finally, 2 mL of this solution was filtered through a PVDF filter (0.45 µM, Millipore) for HPLC analysis.

#### 3.7.3. Preparation of Validated Extracted Samples

This study measured 2.0 g of the powdered *S. thorelii* rhizome into a 50.0 mL glass Erlenmeyer flask, to which 20.0 mL of MeOH was added. The solution was then ultrasonicated three times for 30 min before being filtered and evaporated to about 5.0 mL of a concentrated extract. The concentrated extract was put into a 20.0 mL volumetric flask, rinsed two times with 5.0 mL of MeOH, ultrasonicated, and diluted to 10.0 mL with MeOH. Finally, 2.0 mL of this solution was filtered through a PVDF filter (0.45 µm, Millipore) for HPLC analysis.

### 3.8. Statistical Analysis

The results obtained were displayed as mean ± standard errors (SD). The experiments were conducted in triplicate on three different occasions. Statistical analyses were performed using SPSS software (IBM SPSS® software 20) (SPSS Inc., Chicago, IL, USA). The difference between two or more groups based on one-way of ANOVA multiple comparisons, where *p* < 0.01 indicates statistical significance. Inhibitory concentrations at (IC_50_) were attained from the Sigma plot (12.5) using the nonlinear regression equation log (concentration) versus response–variable slope.

## 4. Conclusions

Bioassay-guided isolation yielded seven compounds from 95% EtOH extract of *S. thorelii,* compound **1** was a new *C*-benzylated dihydrochalcone derivative, compound **3** [(2*E*)-1-(2,4-dihydroxy-6-methoxyphenyl)-3-(2,4-dimethoxyphenyl)-2-propen-1-one] was isolated from a plant for the first time, and this was the first time for the other compounds to be reported in this plant. The effective antiproliferative extracts and compounds yielded from *S. thorelii* through our studies provide scientific evidence to support this plant serving as a folk medicine to reduce tumor size. The bioassay results also revealed that dihydrochalcone derivatives **1** and **3** had promising cytotoxic effects against the HepG2 and WiDr cell lines.

**Figure 5:**
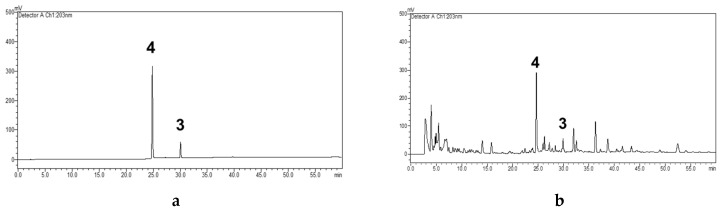
HPLC diagram of specificity. (**a**) Standard sample: 3 and 4 (**b**) *S. thorelii* extract.

Similar to the first report, the contents of the major compounds in *S. thorelii* were determined simultaneously by a simple, accurate, and rapid HPLC-UV data. Compound **3**, the most potent anticancer isolated compound, together with the major yielded isolate, compound **4** [(+)-Crotepoxide], were chosen as analytical markers of this species. The quantification data of **4** and **3,** as shown in Figure 5 and Table 5, concluded that the content of these analytical markers was suitable for comparing the titled plants collected in several areas. It is suggested that this validated method could be applied for the quality control of this herbal medicine in the future.

## Figures and Tables

**Table 1:** Antiproliferative effects of the fractions of the rhizomes of *S. thorelii* against four human cancer cell lines.

Sample	IC_50_ (µg/mL)			
	A549	MCF-7	WiDr	HepG2
SEA	72.83 ± 2.42	69.08 ± 1.24	46.81 ± 5.49	43.48 ± 4.84
SF3	>100	>100	43.42 ± 0.55	>100
SF7	10.52 ± 0.51	7.89 ± 0.59	25.49 ± 0.87	76.38 ± 0.47
SF9	13.12 ± 0.62	12.07 ± 1.61	20.04 ± 2.25	46.15 ± 1.42
SF3.5	51.67 ± 4.64	52.09 ± 1.31	25.5 ± 1.15	43.87 ± 2.71
SF3.6	25.18 ± 2.88	64.16 ± 5.08	55.69 ± 1.36	21.29 ± 0.72

The results shown are means ± SD of triplicate experiments.

**Table 2:** ^1^H and ^13^C-NMR data for compound **1** in acetone-*d*_6_ and **2** in DMSO-*d_6_*.

No	1		No	2	
	δ_H_	δ_C_		δ_H_	δ_C_
1		118.1	1		
2		161.2	2	5.55 (dd, 12.5, 2.5)	73.0
3	6.64 (d, 2.0)	99.1	3	2.95 (dd, 16.5, 12.5) 2.67 (dd, 16.5, 2.5)	44.0
4		164.1	4		187.9
5	6.60 (dd, 8.5, 2.0)	107.0	5		164.5
6	7.65 (d, 8.5)	131.2	6	5.95 (d, 2.5)	95.6
7	8.02 (d, 15.5)	126.0	7		164.4
8	8.09 (d, 15.5)	138.5	8	6.06 (d, 2.5)	93.3
9		193.5	9		162.2
10		105.9	10		104.3
11		166.4	1′		119.1
12		108.2	2′		160.7
13		164.4	3′	6.61 (d, 2.0)	105.0
14	6.20 (s)	92.5	4′		157.3
15		162.6	5′	6.57 (dd, 8.0, 2.0)	98.5
16	3.89 (s)	23.2	6’	7.38 (d, 8.0)	127.7
17		128.2	5-OMe	3.73 (s)	55.7
18		155.6	2′-OMe	3.79 (s)	55.3
19	6.80 (dd, 8.0, 2.0)	116.2	4′-OMe	3.78 (s)	55.6
20	6.98 (ddd, 8.0, 7.5, 2.0)	127.7			
21	6.71 (ddd, 8.0, 7.5, 2.0)	120.3			
22	7.27 (dd, 7.5, 2.0)	131.5			
2-OMe	3.95 (s)	56.1			
4-OMe	3.92 (s)	56.2			
15-OMe	3.87 (s)	55.9			

**Table 3:** Antiproliferative effects of the isolates of the rhizome of *S. thorelii* against four human cancer cell lines.

Compound	IC_50_ (µM)			
	A549	MCF-7	WiDr	HepG2
1	19.93 ± 0.59 ^b^	>200	39.05 ± 1.61 ^b^	9.05 ± 1.47 ^b^
2	>200	>200	>200	>200
3	>200	>200	72.09 ± 1.15 ^c^	11.25 ± 0.41 ^b^
4	>200	>200	>200	77.74 ± 5.28 ^c^
5	94.54 ± 0.45 ^c^	>200	>200	>200
6	>200	>200	>200	153.25 ± 5.74 ^d^
Mitomycin *c*	0.32 ± 0.03 ^a^	0.5 ± 0.03	0.56 ± 0.03 ^a^	0.29 ± 0.03 ^a^

The results shown are means ± SD of triplicate experiments. The same superscript letters in a same column show nonsignificant differences among samples at *p* < 0.01.

**Table 4:** Quantification of compounds **4** and **3** in the rhizome of *S. thorelii* from different collection areas.

Place to Collect	Collected Date	4 (%)	3 (%)
An Giang province	01/12/2018	0.109	0.013
Ho Chi Minh city	02/12/2018	0.106	0.012
Dong Nai province	02/12/2018	0.102	0.013
Average		0.106	0.013

**Table 5:** The result of the validation of HPLC method for quantitative analysis of compounds **4** and **3**.

Validation Criteria		4	3
Linearity			
Regression equation		Y = 32638X	Y = 57440X
Linear range (µg/mL)		2.5–500	2.5–500
R^2^ (≤2)		0.9956	0.9969
LOD		0.05 µg/mL	0.025 µg/mL
LOQ		0.17 µg/mL	0.08 µg/mL
System suitability			
R^2^-t_R_ (≤2)		0.1443	0.0676
R^2^–Area (≤2)		0.1358	1.2298
R^2^–T. plate (≤2)		1.5610	1.1262
R^2^–Resolution (≤2)		1.8971	1.7550
*Repeatability* (*n* = 6)			
R^2^-t_R_ (≤2)		0.1469	0.1136
R^2^–Area (≤5.3)		3.3516	2.5904
Accuracy			
Level 1 (*n* = 3)	% recovery	96.55	96.12
	R^2^ (≤5.3)	2.74	5.15
Level 2 (*n* = 3)	% recovery	95.8	87.50
	R^2^ (≤5.3)	3.00	4.25
Level 3 (*n* = 3)	% recovery	94.05	97.50
	R^2^ (≤5.3)	2.58	4.01

## Supplementary Materials

The following are available online at https://www.mdpi.com/1420-3049/25/3/551/s1.

## Abbreviations

A549	human lung adenocarcinoma
CAN	acetonitrile
ATCC	American type culture collection
CDCl_3_	chloroform
DMSO	dimethyl sulfoxide
HepG2	human liver adenocarcinoma
HPLC	high performance liquid chromatography
HR-ESI-MS	high resolution electrospray ionization mass spectrometry
IC50	50% inhibitory concentration
MCF-7	human breast adenocarcinoma
WiDr	human colon adenocarcinoma
MTT	3-(4,5-dimethylthiazol-2-yl)-2,5-diphenyl tetrazolium
MEM	minimum essential medium
NMR	nuclear magnetic resonance
OD	optical density
PBS	phosphate buffered saline
TLC	thin-layer chromatography
MeOH	methanol
COSY	correlation spectroscopy
HMBC	heteronuclear multiple bonds correlation
NOESY	nuclear Overhauser effect spectroscopy
DEPT	distortionless enhancement by polarization transfer
FBS	fetal bovine serum
m/z	mass to charge ratio
Hz	hertz
CC	column chromatography
HSQC	homonuclear single quantum correlation
UV	ultraviolet spectroscopy
ICH	International Conference Harmonization
PVDF	polyvinylidene fluoride
RSD	relative standard deviation
IR	Infrared spectroscopy
HT29	human colon adenocarcinoma
B164A5	murine melanoma
JAK/STAT	Janus kinase/signal transducers and activators of transcription
EtOAc	ethyl acetate
CD	circular dichroism
SPD	photodiode array detector
ANOVA	analysis of variance

## References

[1] Lu C.L., Zhao H.Y., Jiang J.G. (2013). Evaluation of multi-activities of 14 edible species from Zingiberaceae. Int. J. Food Sci. Nutr..

[2] Kirana C., Record I.R., McIntosh G.H., Jones G.P. (2003). Screening for antitumor activity of 11 species of indonesian zingiberaceae using human MCF-7 and HT-29 cancer cells. Pharma. Biol..

[3] Danciu C., Vlaia L., Fetea F., Hancianu M., Coricovac D.E., Ciurlea S.A., Şoica C.M., Marincu I., Vlaia V., Dehelean C.A. (2015). Evaluation of phenolic profile, antioxidant and anticancer potential of two main representants of Zingiberaceae family against B164A5 murine melanoma cells. Biol. Res..

[4] Rao K.V., Samikkannu T., Dakshayani K.B., Zhang X., Sathaye S.S., Indap M.A., Nair M.P. (2012). Chemopreventive potential of an ethyl acetate fraction from Curcuma longa is associated with up-regulation of p57kip2 and Rad9 in the PC3M prostate cancer cell line. Asian Pac. J. Cancer Prev..

[5] Cheng X.L., Liu Q., Peng Y.B., Qi L.W., Li P. (2011). Steamed ginger (Zingiber officinale): Changed chemical profile and increased anticancer potential. Food Chem..

[6] Park G.H., Park J.H., Song H.M., Eo H.J., Kim M.K., Lee J.W., Lee M.H., Cho K.H., Lee J.R., Cho H.J. (2014). Anti-cancer activity of Ginger (Zingiber officinale) leaf through the expression of activating transcription factor 3 in human colorectal cancer cells. BMC Complement. Altern. Med..

[7] Kirana C., Record I.R., Jones G.P., McIntosh G.H. (2007). Anticancer properties of panduratin A isolated from *Boesenbergia pandurata* (Zingiberaceae). J. Nat. Med..

[8] Muangnoi P., Lu M., Lee J., Thepouyporn A., Mirzayans R., Le X.C., Weinfeld M., Changbumrung S. (2007). Cytotoxicity, Apoptosis and DNA Damage Induced by *Alpinia galangal* Rhizome Extract. Planta Med..

[9] Li Y., Sun W., Han N., Zou Y., Yin D. (2018). Curcumin inhibits proliferation, migration, invasion and promotes apoptosis of retinoblastoma cell lines through modulation of miR-99a and JAK/STAT pathway. BMC Cancer.

[10] Li Q.M., Luo J.G., Wang R.Z., Wang X.B., Yang M.H., Luo J., Kong L.Y. (2016). Involucratusins A-H: Unusual Cadinane Dimers from Stahlianthus involucratus with Multidrug Resistance Reversal Activity. Sci. Rep..

[11] Vo V.C. (2012). Tu dien cay thuoc Viet Nam-Vietnamese Herbal Medicine Dictionary.

[12] Yu J.G., Chen Y.H., Fang H.J. (1983). Studies on the medicinal plants of Chinese Zingiberaceae. Acta. Pharm. Sin..

[13] Li Q.M., Luo J.G., Zhang Y.M., Li Z.R., Wang X.B., Yang M.H., Luo J., Sun H.B., Chen Y.J., Kong L.Y. (2015). Involucratustones A-C: Unprecedented sesquiterpene dimers containing multiple contiguous quaternary carbons from stahlianthus involucratus. Chemistry.

[14] Pingsusaen P., Kunanusorn P., Khonsung P., Chiranthanut N., Panthong A., Rujjanawate C. (2015). Investigation of anti-inflammatory, antinociceptive and antipyretic activities of Stahlianthus involucratus rhizome ethanol extract. J. Ethnopharmacol..

[15] Nagarajan G.R., Parmar V.S. (1977). Three new flavonoids in Prunus cerasus. Phytochemisty.

[16] Aponte J.C., Castillo D., Estevez Y., Gonzalez G., Arevalo J., Hammond G.B., Sauvain M. (2010). In vitro and in vivo anti-Leishmania activity of polysubstituted synthetic chalcones. Bioorg. Med. Chem. Lett..

[17] Makhuvele R., Foubert K., Apers S., Pieters L., Verschaeve L., Elgorashi E. (2018). Antimutagenic constituents from Monanthotaxis caffra (Sond.) Verdc. J. Pharm. Pharmacol..

[18] Montree Kodpinida C.S. (1983). Chachanat Thebtaranonth, Yodhathai Thebtaranont, Structures of β-senepoxide, tingtanoxide, and their diene precursors. Constituents of Uvaria ferruginea. Tetrahedron Lett..

[19] Awale S., Ueda J.Y., Athikomkulchai S., Abdelhamed S., Yokoyama S., Saiki I., Miyatake R. (2012). Antiausterity agents from Uvaria dac and their preferential cytotoxic activity against human pancreatic cancer cell lines in a nutrient-deprived condition. J. Nat. Prod..

[20] Muto N., Tomokuni T., Haramoto M., Tatemoto H., Nakanishi T., Inatomi Y., Murata H., Inada A. (2008). Isolation of apoptosis- and differentiation-inducing substances toward human promyelocytic leukemia HL-60 cells from leaves of Juniperus taxifolia. Biosci. Biotechnol. Biochem..

[21] Awale S., Tawila A.M., Dibwe D.F., Ueda J.Y., Sun S., Athikomkulchai S., Balachandran C., Saiki I., Matsumo K., Esumi H. (2017). Highly oxygenated antiausterity agents from the leaves of Uvaria dac. Bioorg. Med. Chem. Lett..

[22] Lekphrom R., Kanokmedhakul K., Schevenels F., Kanokmedhakul S. (2018). Antimalarial polyoxygenated cyclohexene derivatives from the roots of Uvaria cherrevensis. Fitoterapia.

