# Supplementary material for: Bioassay-Guided Isolation and HPLC Quantification of Antiproliferative Metabolites from Stahlianthus thorelii
Source: Molecules. 2020 Jan 28;25(3):551. doi: 10.3390/molecules25030551 (PMC7038115; doi:10.3390/molecules25030551)
Supplement: Supplementary file 1 [file molecules-25-00551-s001.pdf]

SUPPLEMENT MATERIAL

Bioassay-guided isolation and HPLC quantification of antiproliferative metabolites from *Stahlianthus thorelii*

Nham-Linh Nguyen[a,b], Hoa Thanh Vo[a,b], Yu-Chi Lin[b], Zhi-Hu Lin[b], Mei-Chuan Chen[a*], and Yao-Haur Kuo[b,c*]

^{1}H NMR and ^{13}C-NMR spectrums, and HREIMS of *Stahlianthus thorelii*

1. ^{1}H NMR and ^{13}C-NMR spectrums, and HREIMS of (E)-1-(3',5'-dihydroxy-4'-(2''-hydroxybenzyl) -2'- methoxyphenyl) -3- (2,4-dimethoxyphenyl) prop-2-en-1-one (1)

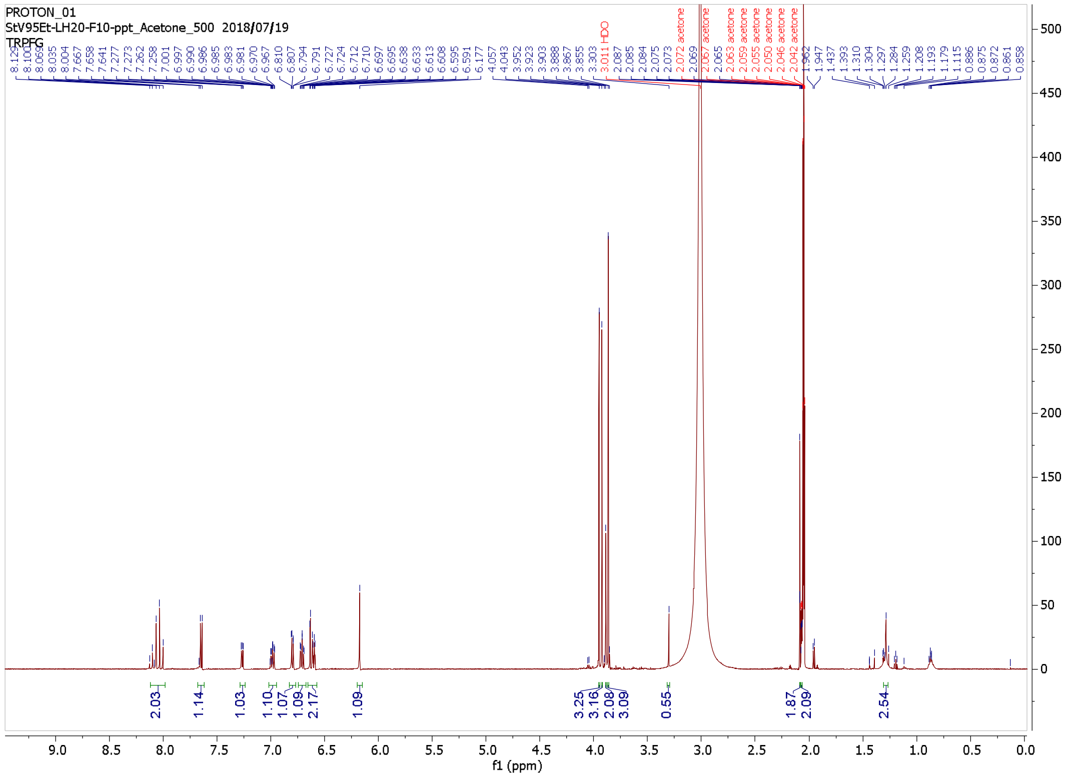

Figure S1. ^{1}H NMR spectrum of ((E)-1-(3',5'-dihydroxy-4'-(2''- hydroxybenzyl) -2'-methoxyphenyl) -3- (2,4-dimethoxyphenyl) prop-2-en-1-one) **(1)** measured in 500 MHz in acetone

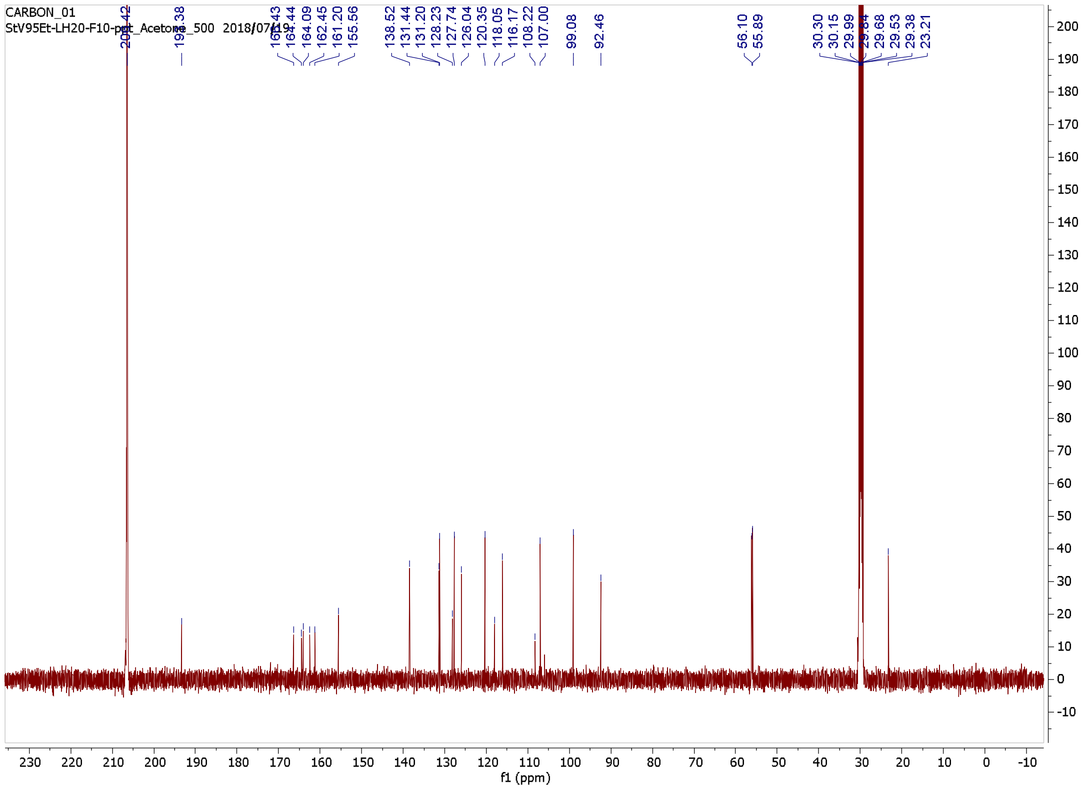

Figure S2. ^{13}C NMR spectrum of ((E)-1-(3’,5’-dihydroxy-4’-(2’’- hydroxybenzyl) -2’-methoxyphenyl) -3- (2,4-dimethoxyphenyl) prop-2-en-1-one) **(1)** measured in 500 MHz in acetone

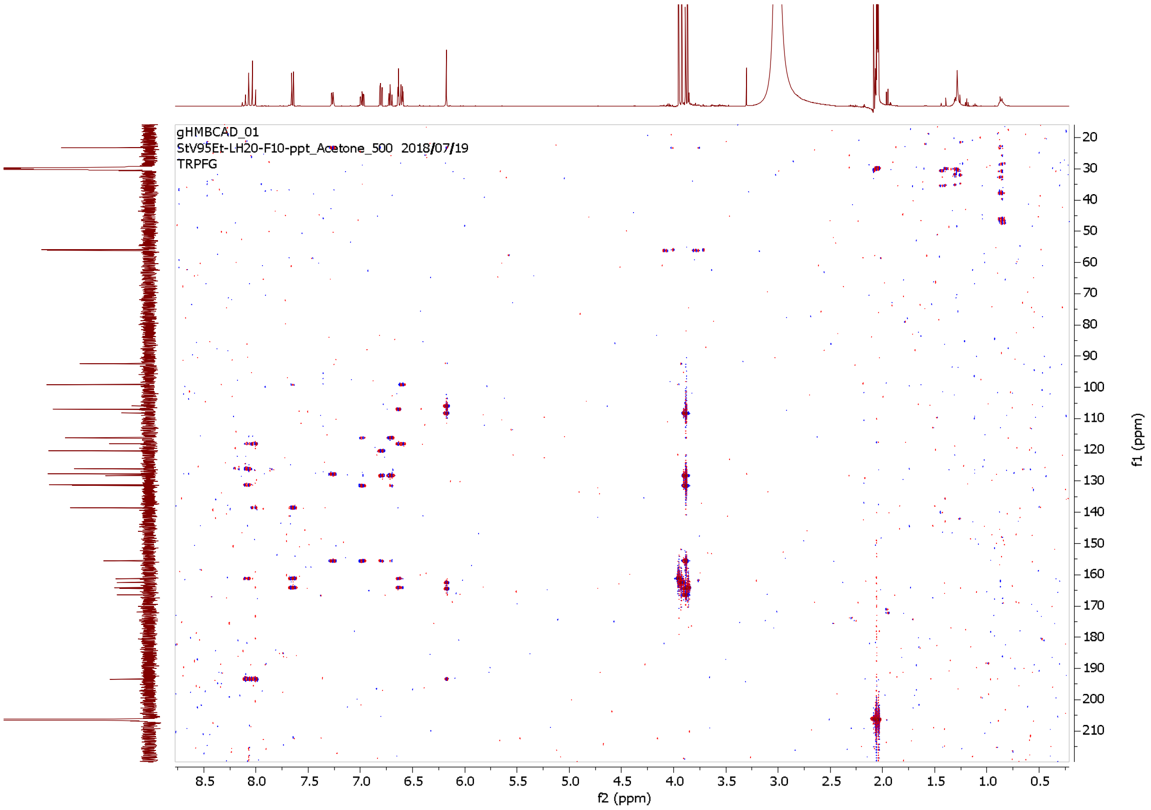

Figure S3. HMBC spectrum of ((E)-1-(3’,5’-dihydroxy-4’-(2’’- hydroxybenzyl) -2’- methoxyphenyl) -3- (2,4-dimethoxyphenyl) prop-2-en-1-one) **(1)** measured in 500 MHz in acetone

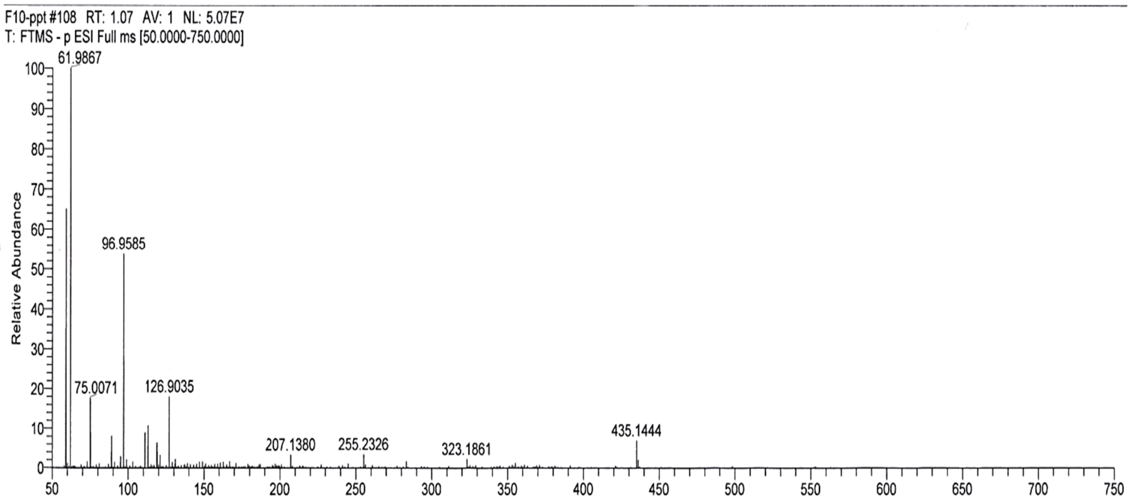

Figure S4. The HREIMS of (E)-1-(3’,5’-dihydroxy-4’-(2’’- hydroxybenzyl) -2’- methoxyphenyl) -3- (2,4-dimethoxyphenyl) prop-2-en-1-one **(1)**, M= 436, $[M - H]^-$

2. ^{1}H NMR and ^{13}C-NMR spectrums, and HREIMS of (2E)-1-(2,4-Dihydroxy-6-methoxyphenyl)-3-(2,4-dimethoxyphenyl)-2-propen-1-one (2)

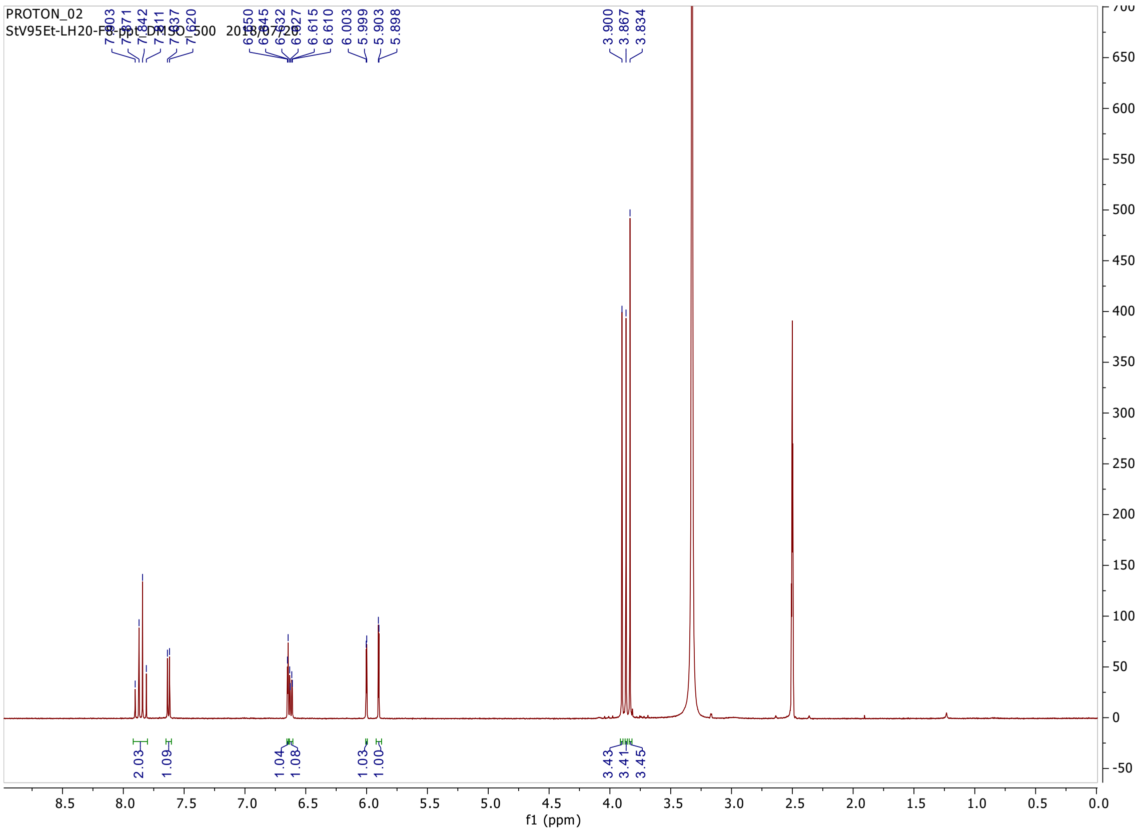

Figure S5. ^{1}H NMR spectrum of (2E)-1-(2,4-Dihydroxy-6-methoxyphenyl)-3-(2,4-dimethoxyphenyl)-2-propen-1-one **(2)** measured in 500 MHz in DMSO

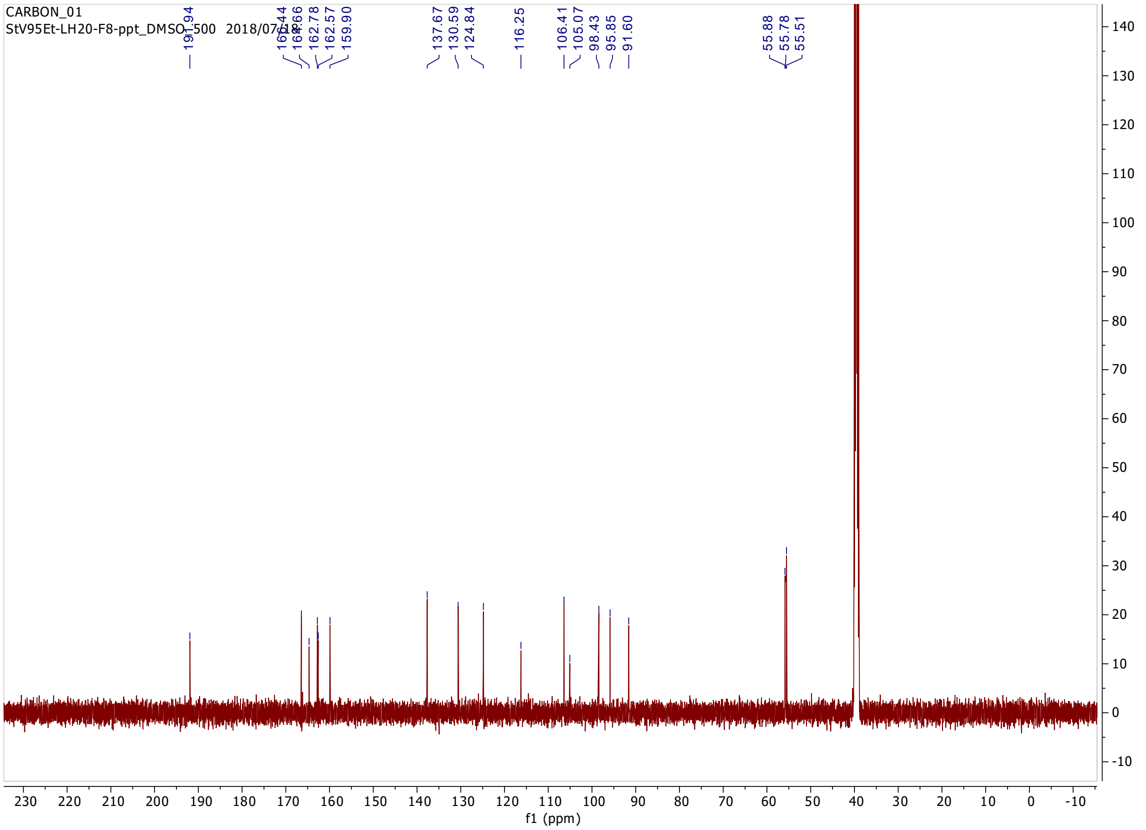

Figure S6. ^{13}C NMR spectrum of (2E)-1-(2,4-Dihydroxy-6-methoxyphenyl)-3-(2,4-dimethoxyphenyl)-2-propen-1-one **(2)** measured in 500 MHz in DMSO

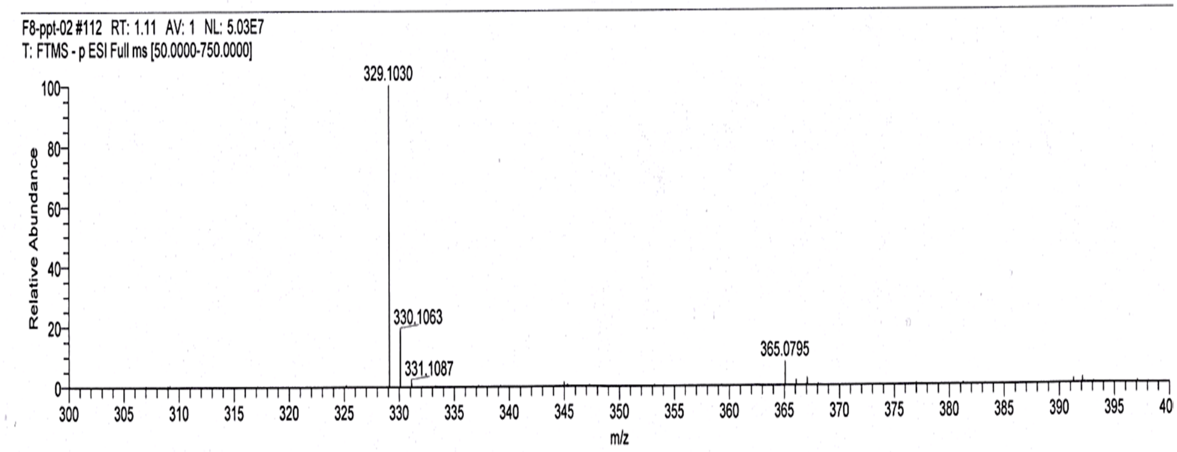

Figure S7. The HREIMS of (2E)-1-(2,4-Dihydroxy-6-methoxyphenyl)-3-(2,4-dimethoxyphenyl)-2-propen-1-one **(2)**, M= 330, $[M - H]^-$

3. ^{1}H NMR and ^{13}C-NMR spectrums, and HREIMS of 7-hydroxyethyloxy-3',4',5-trimethoxy flavone (3)

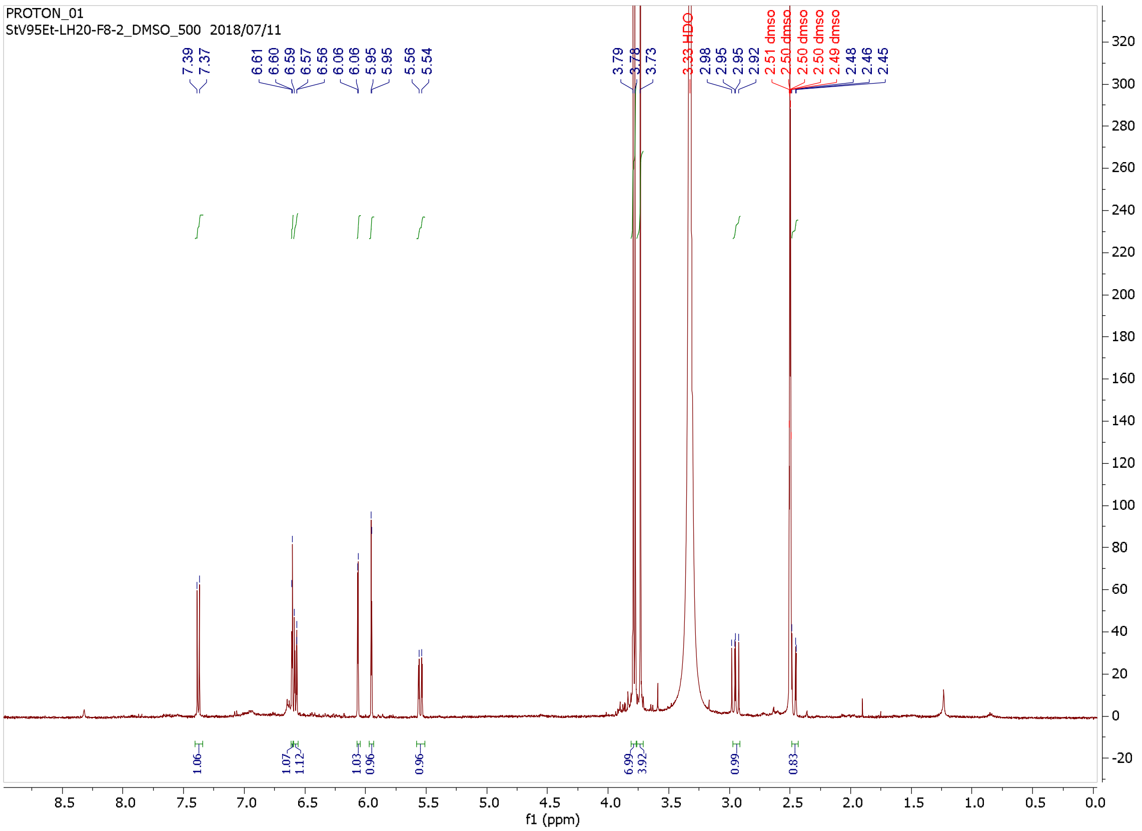

Figure S8. ^{1}H NMR spectrum of 7-hydroxyethyloxy-3',4',5-trimethoxy flavone **(3)** measured in 500 MHz in DMSO

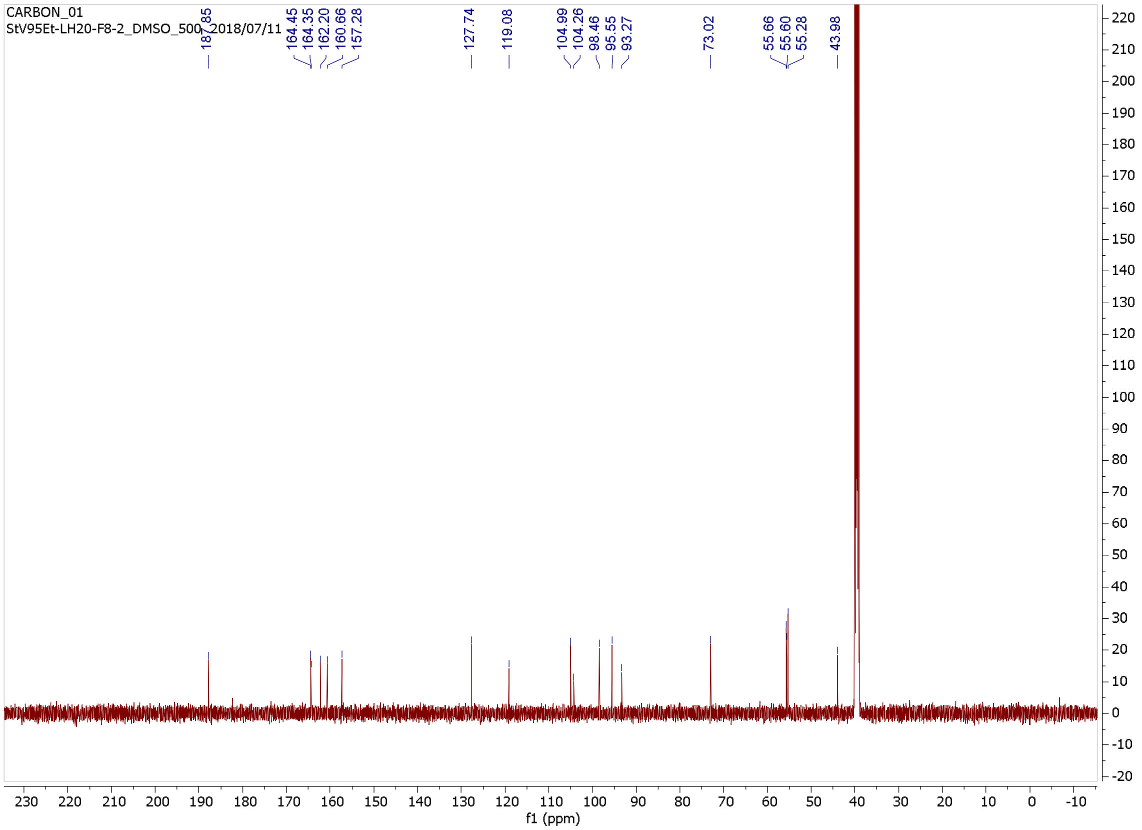

Figure S9. ^{13}C NMR spectrum of 7-hydroxyethyloxy-3',4',5-trimethoxy flavone **(3)** measured in 500 MHz in DMSO

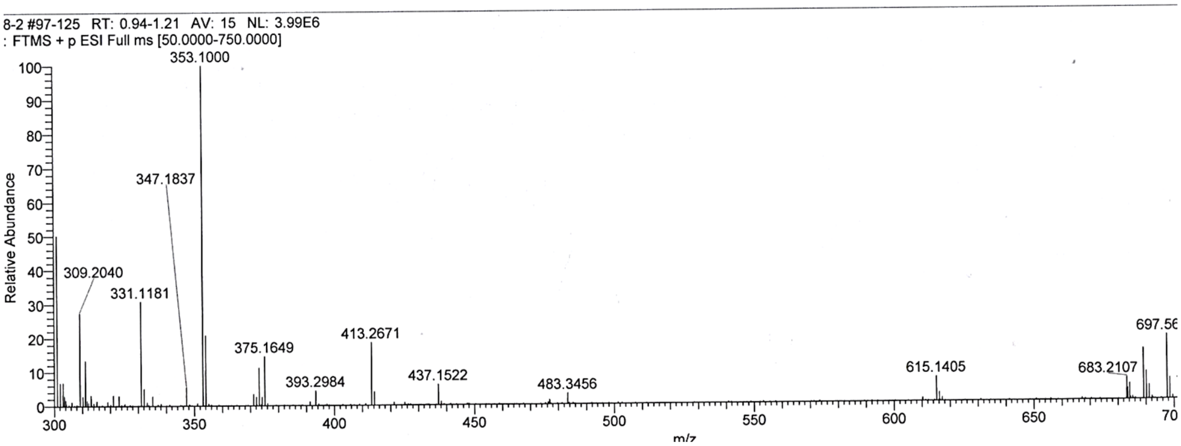

Figure S10. The HREIMS of 7-hydroxyethyloxy-3',4',5-trimethoxy flavone **(3)**, M= 330, [M + Na]$^+$

4. ^{1}H NMR and ^{13}C-NMR spectrums, and HREIMS of (+)-crotepoxide (4)

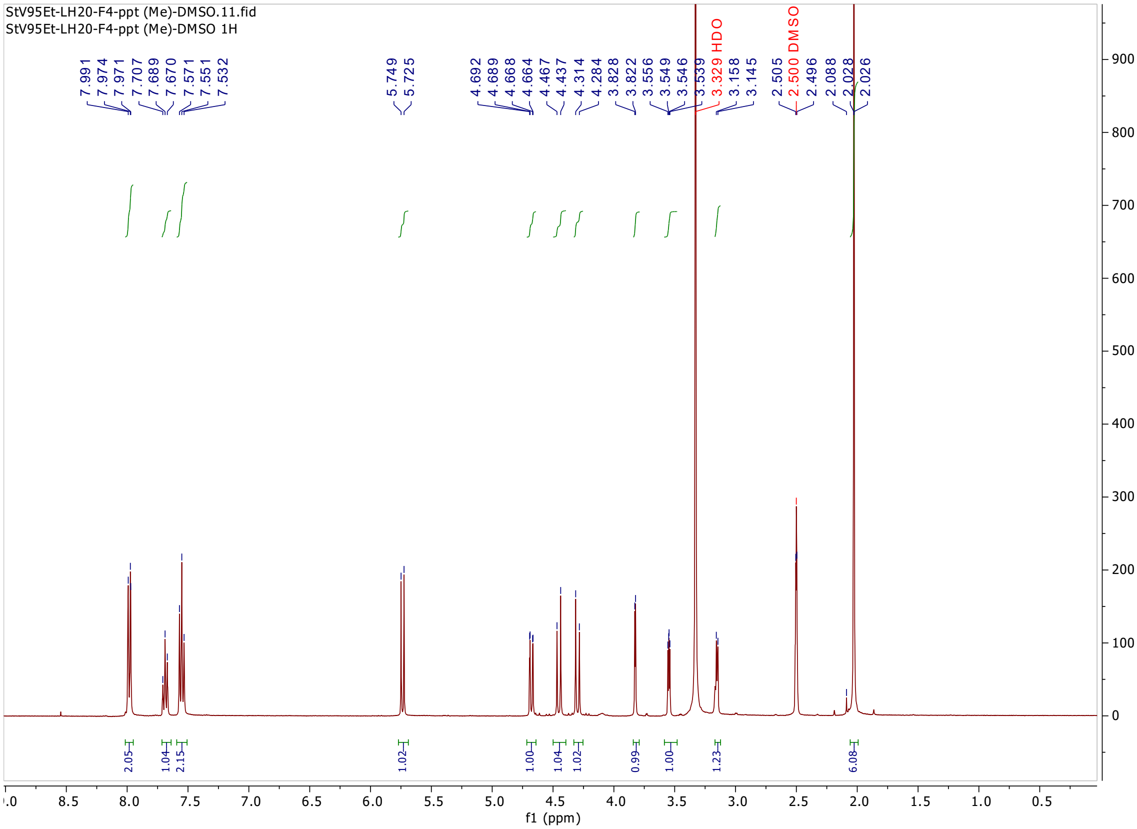

Figure S11. ^{1}H NMR spectrum of (+)-crotepoxide **(4)** measured in 500 MHz in DMSO

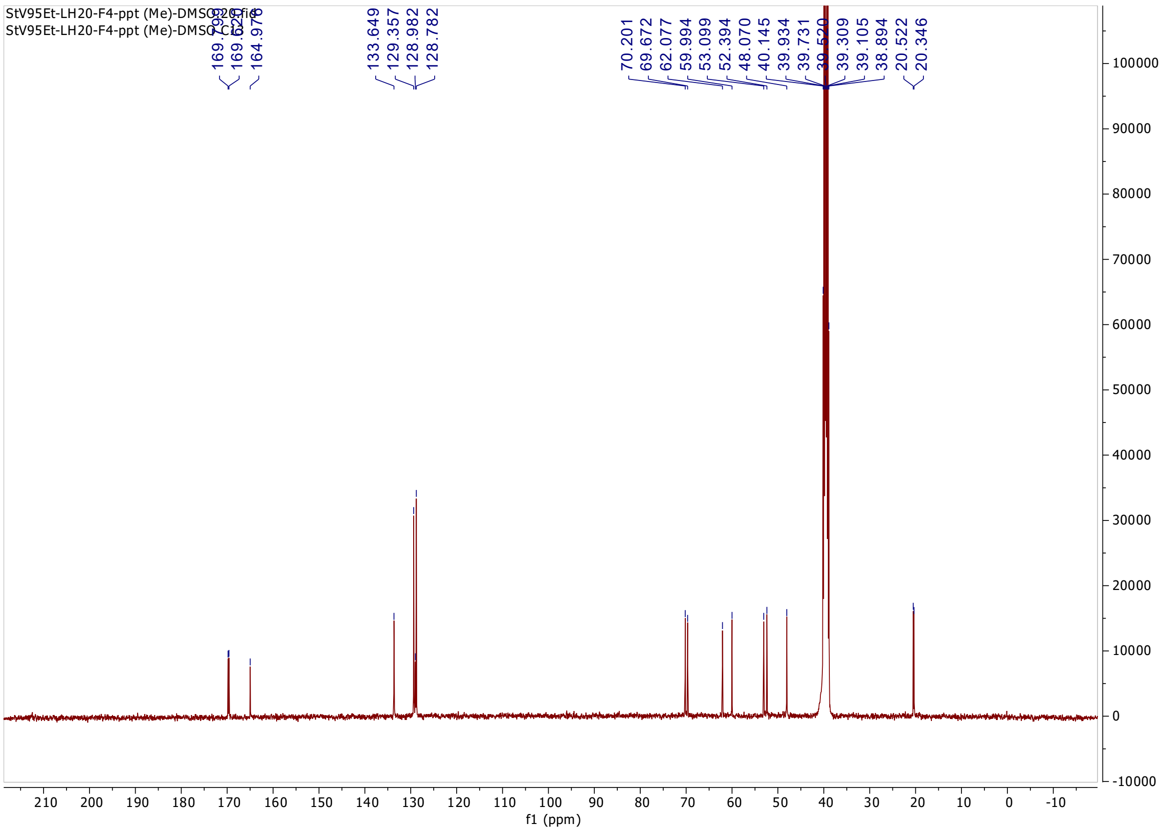

Figure S12. ^{13}C NMR spectrum of (+)-crotepoxide **(4)** measured in 500 MHz in DMSO

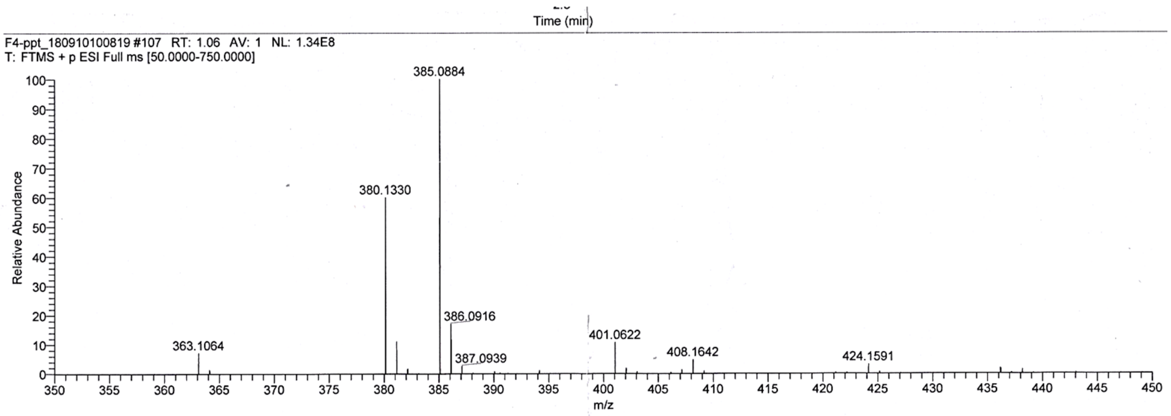

Figure S13. The HREIMS of (+)-crotepoxide **(4)**, M= 362, [M + Na]$^{+}$

5. ^{1}H NMR and ^{13}C-NMR spectrums, and HREIMS of sandaracopimaric acid (5)

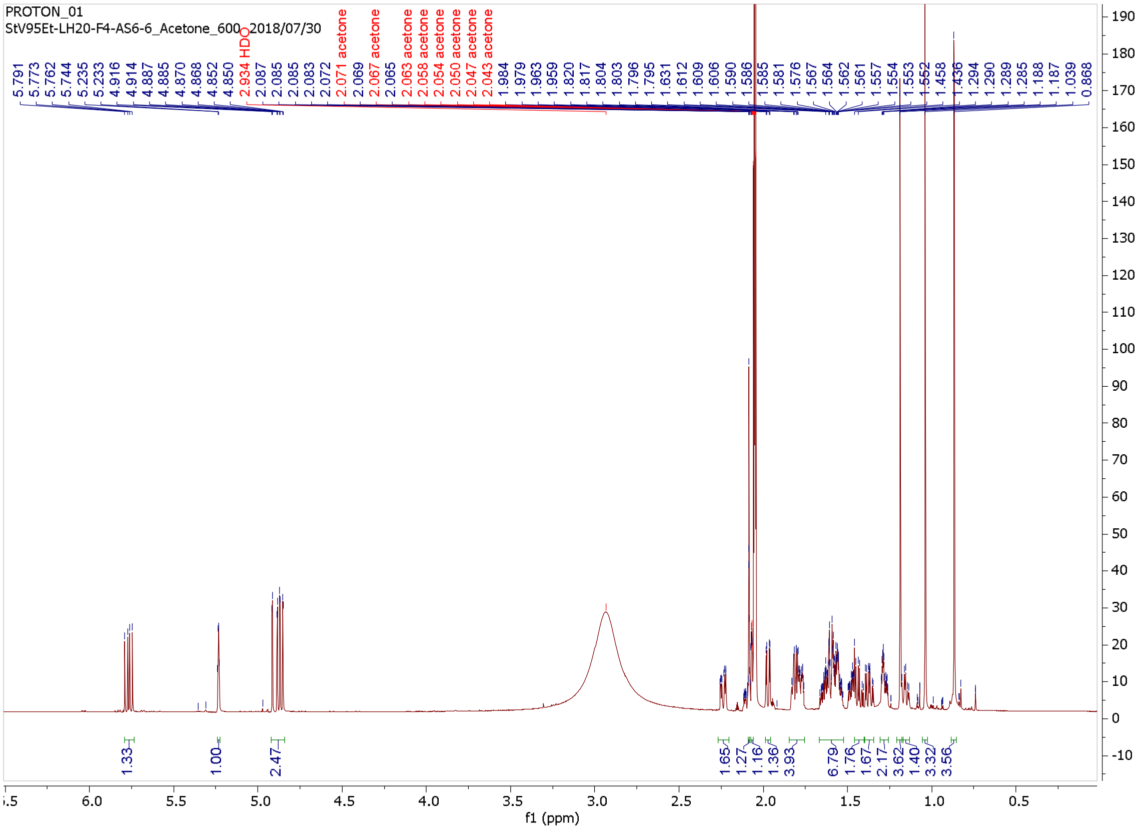

Figure S14. ^{1}H NMR spectrum of sandaracopimaric acid **(5)** measured in 500 MHz in acetone

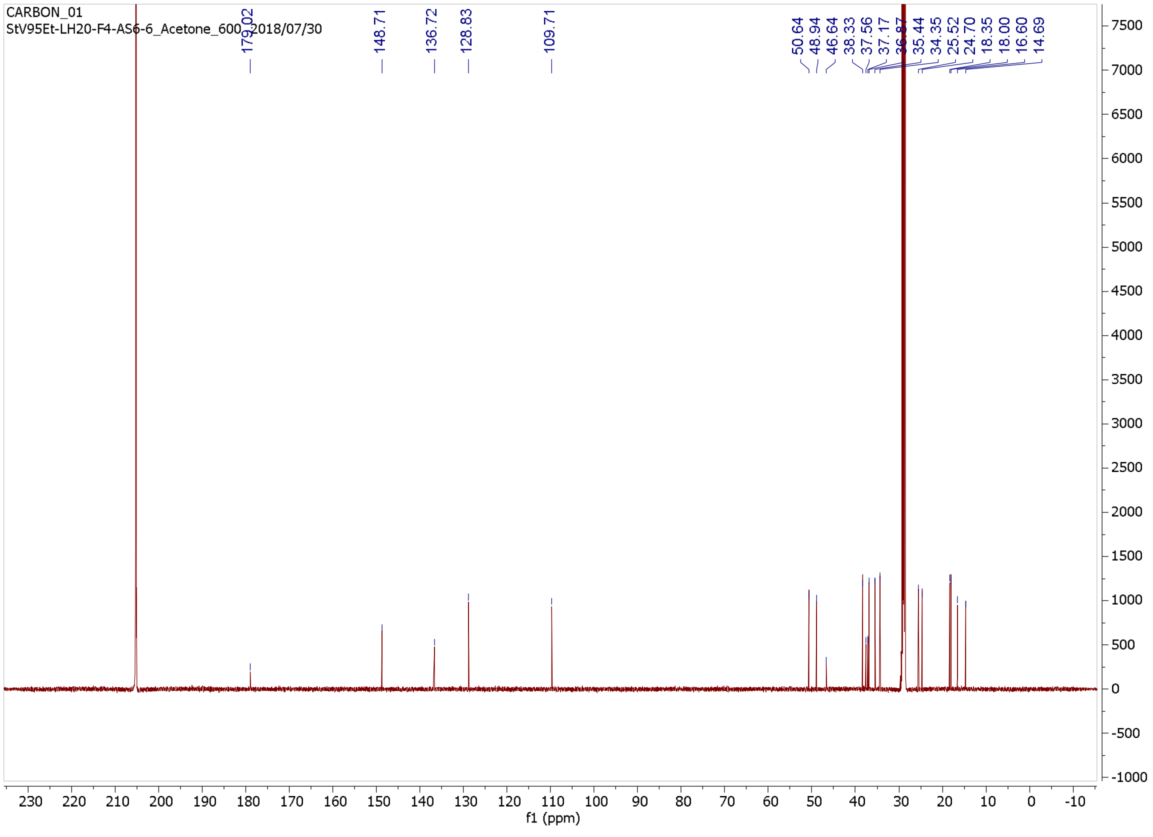

Figure S15. ^{13}C NMR spectrum of sandaracopimaric acid **(5)** measured in 500 MHz in acetone

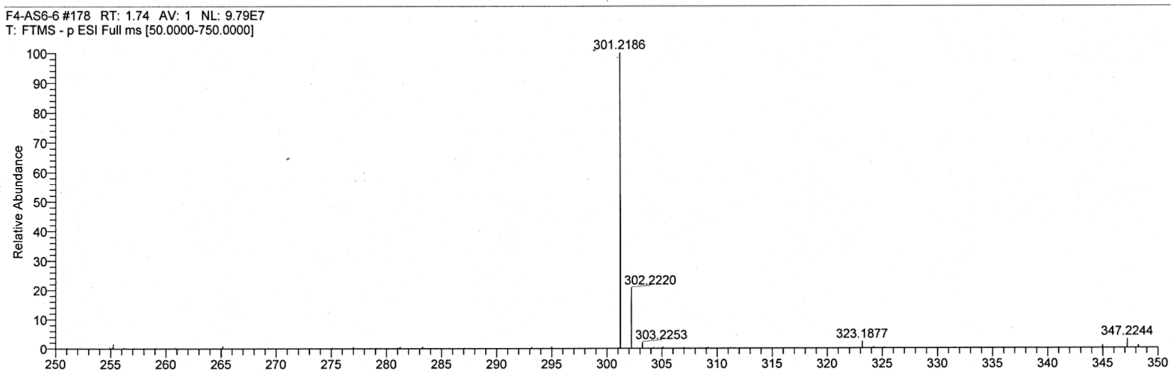

Figure S16. The HREIMS of sandaracopimaric acid **(5)**, M= 302, [M - H]-

6. ^{1}H NMR and ^{13}C-NMR spectrums, and HREIMS of o-methoxybenzoyl benzoate (6)

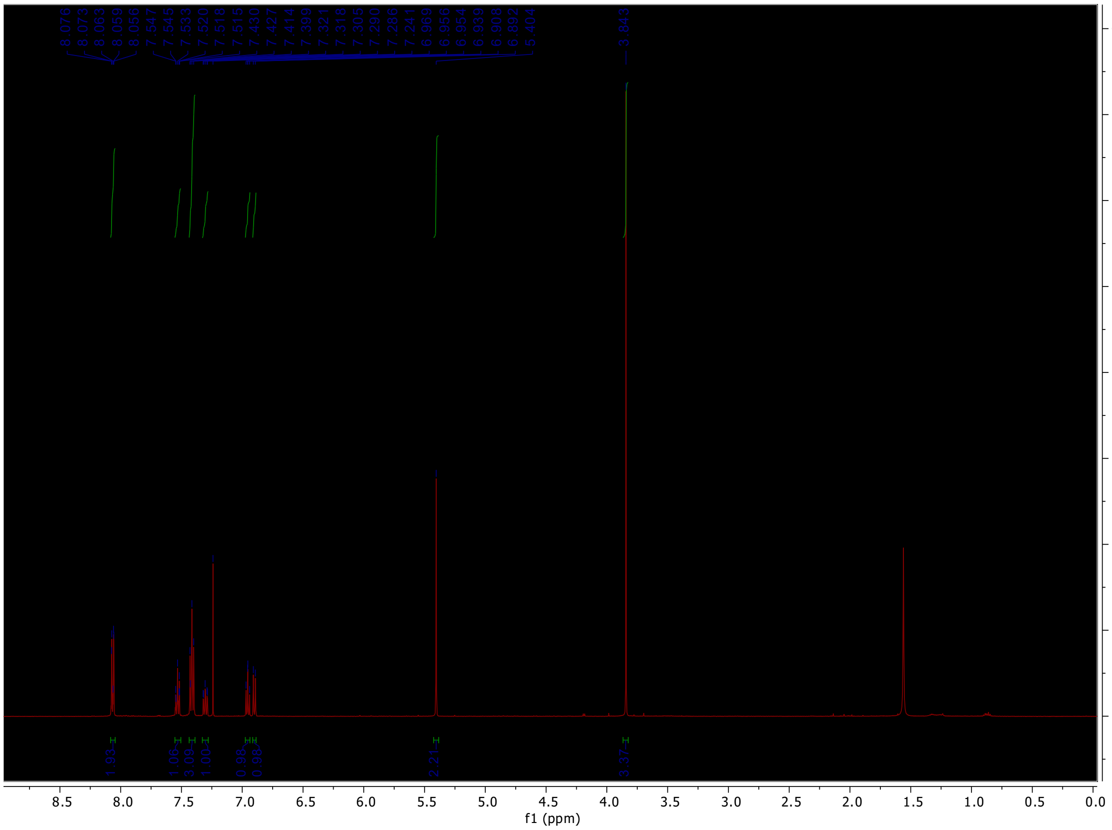

Figure S17. ^{1}H NMR spectrum of o-methoxybenzoyl benzoate **(6)** measured in 500 MHz in $CDCl_3$

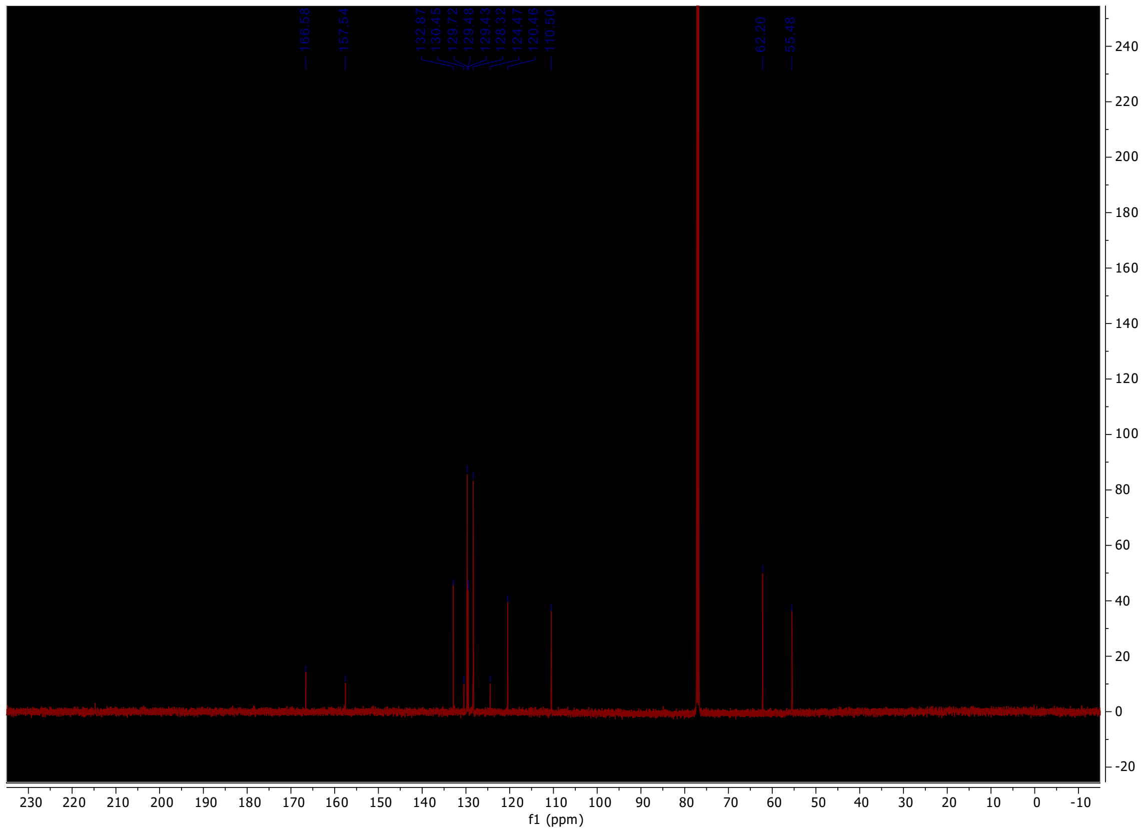

Figure S18. ^{13}C NMR spectrum of o-methoxybenzoyl benzoate **(6)** measured in 500 MHz in $CDCl_3$

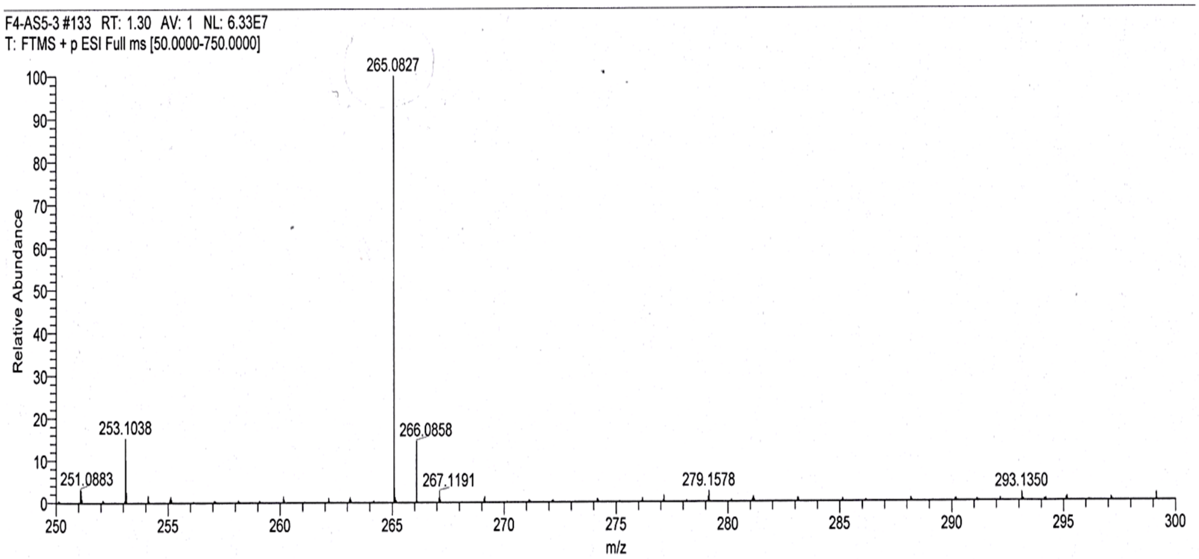

Figure S19. The HREIMS of o-methoxybenzoyl benzoate **(6)**, M= 242 [M + Na]+

7. ^{1}H NMR and ^{13}C-NMR spectrums, and HREIMS of (−)1,6-desoxytingtanoxide (7)

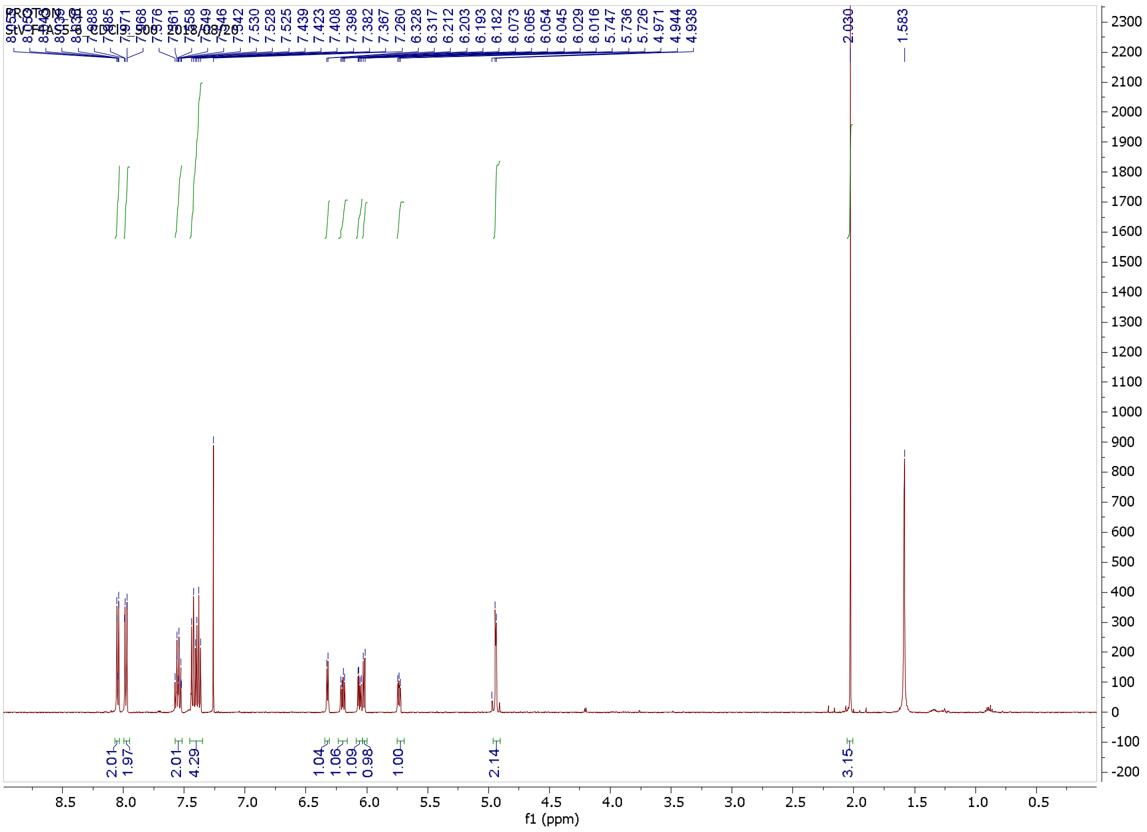

Figure S20. ^{1}H NMR spectrum of (−)1,6-desoxytingtanoxide **(7)** measured in 500 MHz in $CDCl_3$

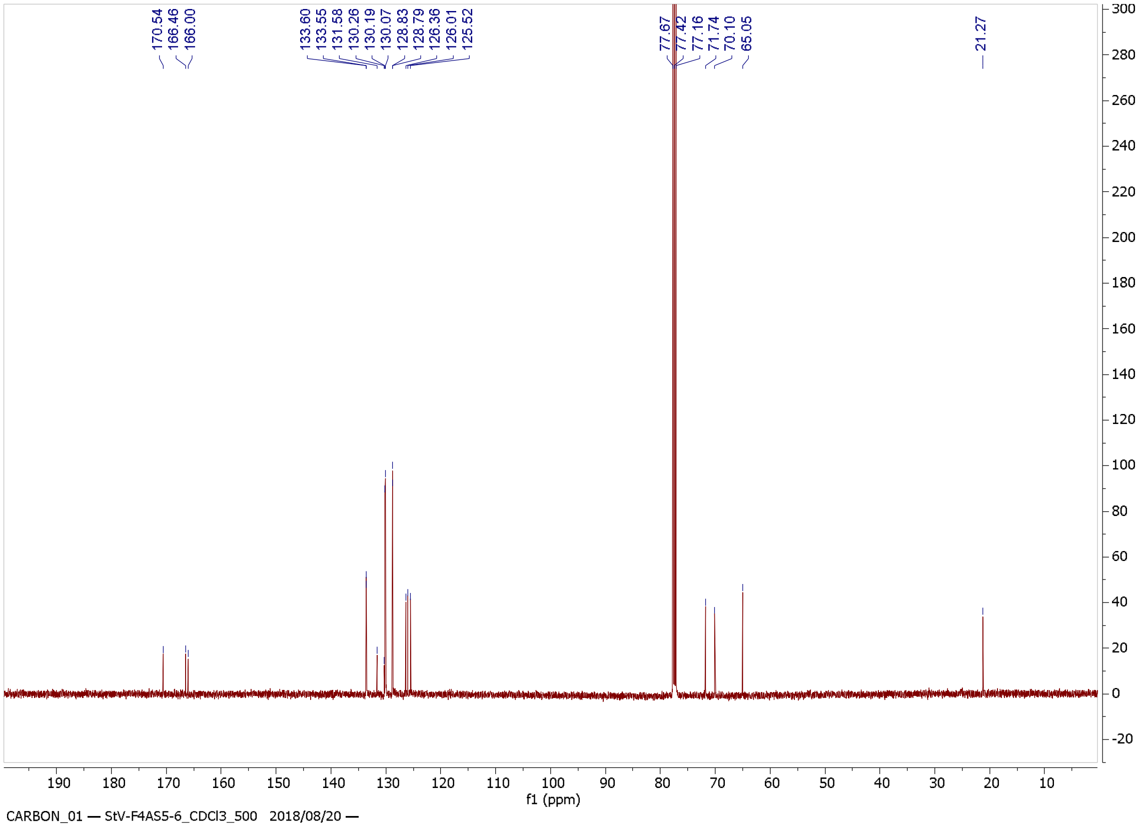

Figure S21. ^{13}C NMR spectrum of (−)1,6-desoxytingtanoxide **(7)** measured in 500 MHz in $CDCl_3$

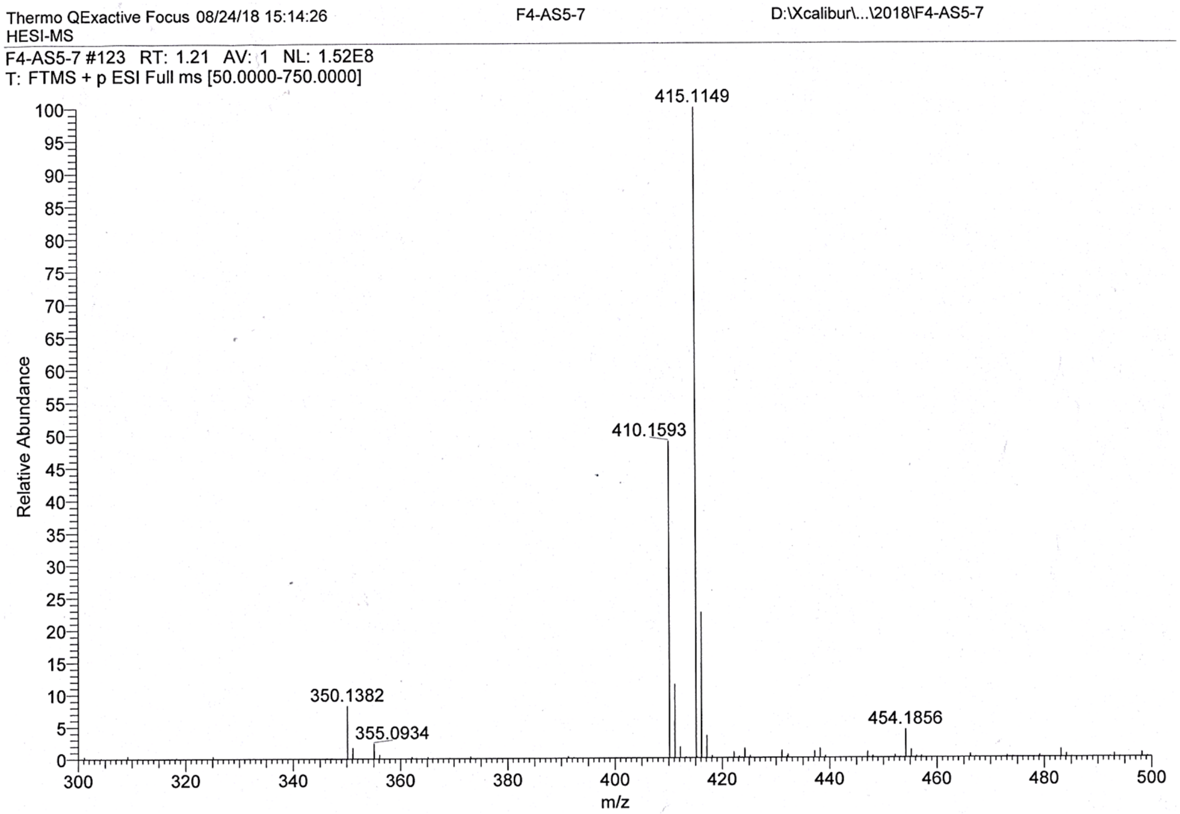

Figure S22. The HREIMS of (−)1,6-desoxytingtanoxide **(7)**, M= 392, [M + Na]+
